# Supplementary figures and images for: Association of social vulnerability factors with power outage burden in Washington state: 2018–2021
Source: PLoS One. 2024 Sep 4;19(9):e0307742. doi: 10.1371/journal.pone.0307742 (PMC11373849; doi:10.1371/journal.pone.0307742)

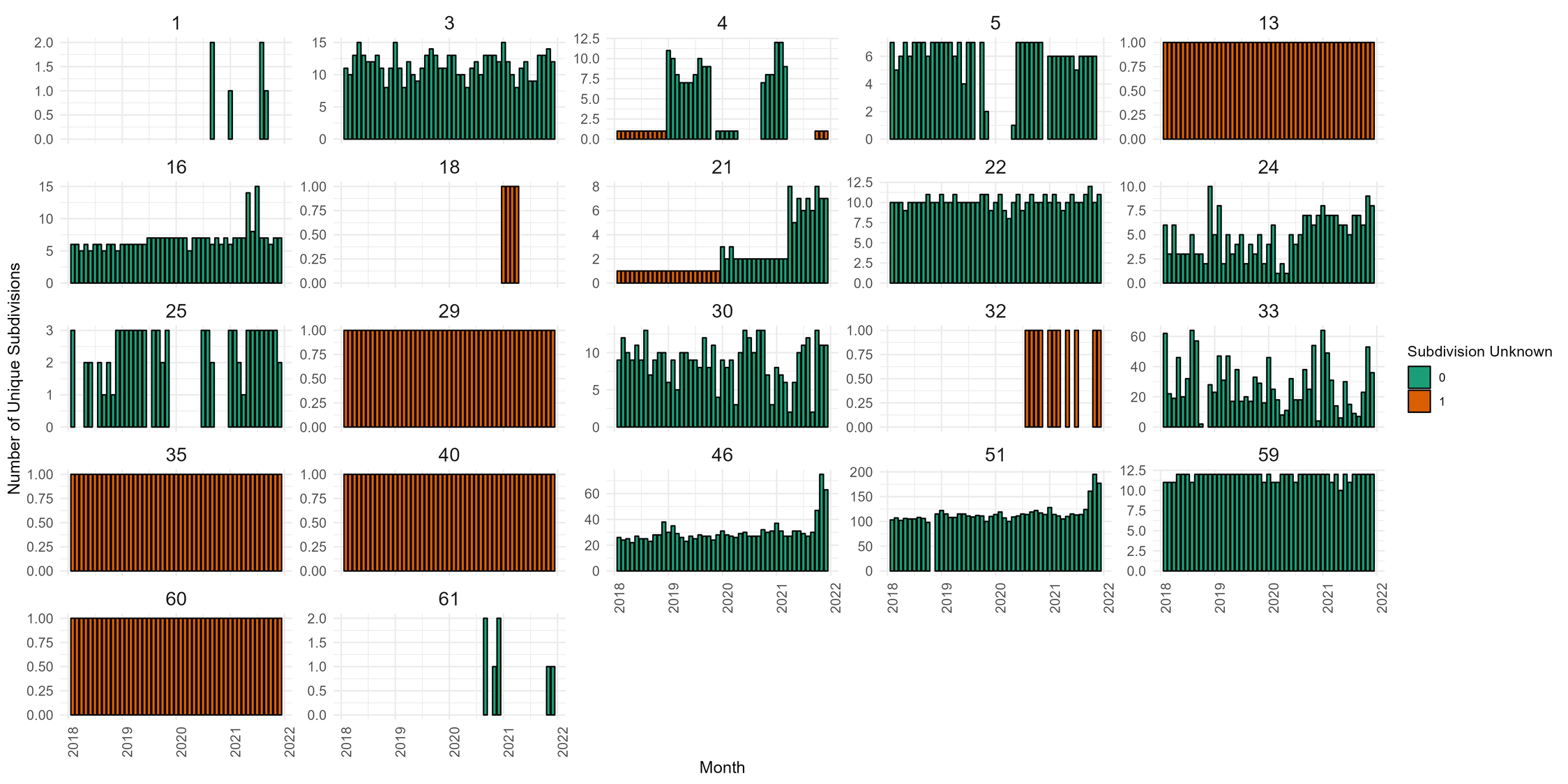

Supplement: S1 Fig — Subdivisions are unknown when outage data is reported at the level of the county. Each panel corresponds to a utility, represented by an anonymized ID. (TIF) [file pone.0307742.s001.tif]

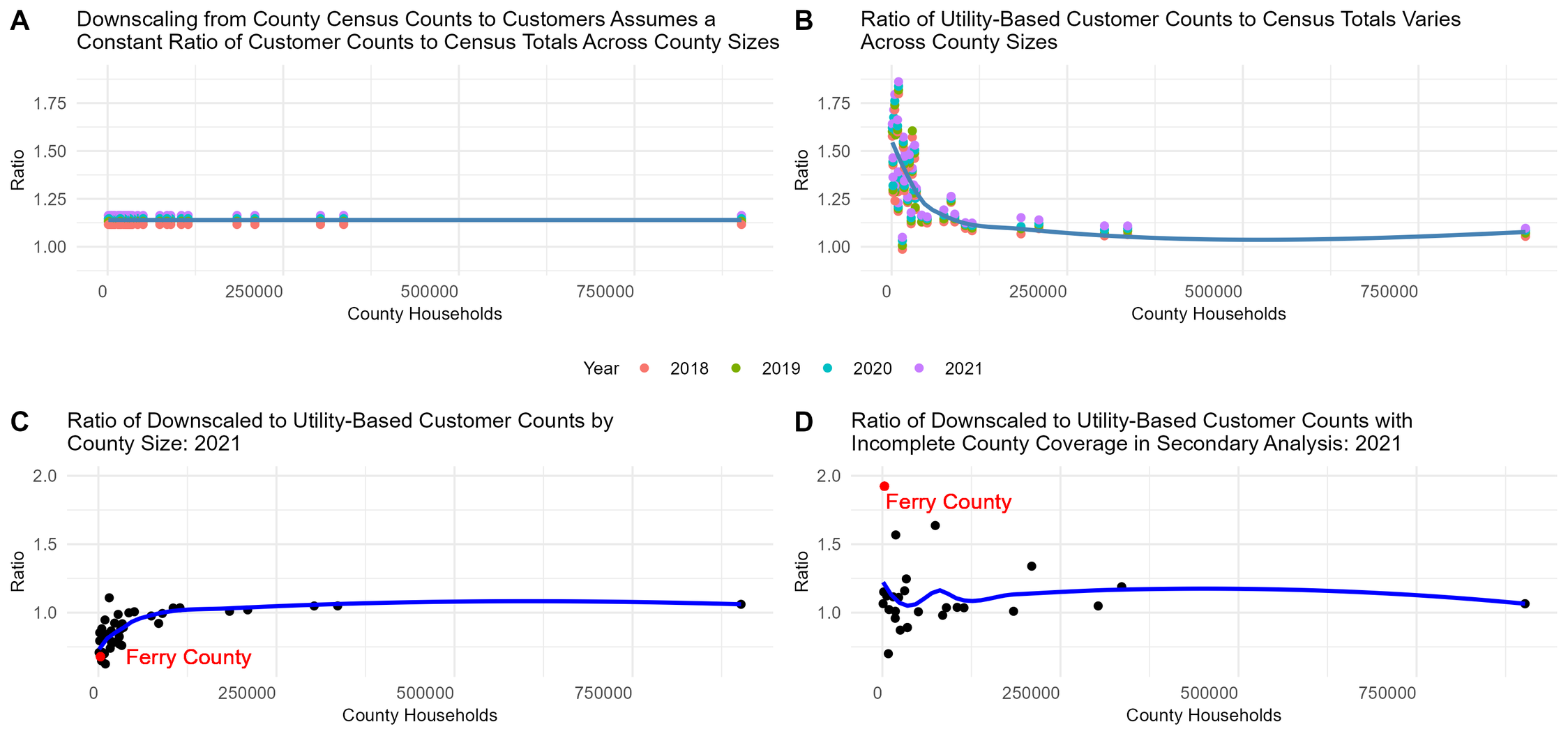

Supplement: S3 Fig — (A) The ratio of downscaled census-based customer counts to census totals (households and establishments) versus households for Washington counties. (B) The ratio of utility-based customer estimates to census totals (households and establishments) versus the number of county households for Washington counties. (C) The ratio of downscaled to utility-based customer counts for the year 2021, with red point for Ferry County. (D) The ratio of downscaled to utility-based customer counts summed for utilities included in PowerOutage.us data for the year 2021, with red point for Ferry County. (TIF) [file pone.0307742.s003.tif]

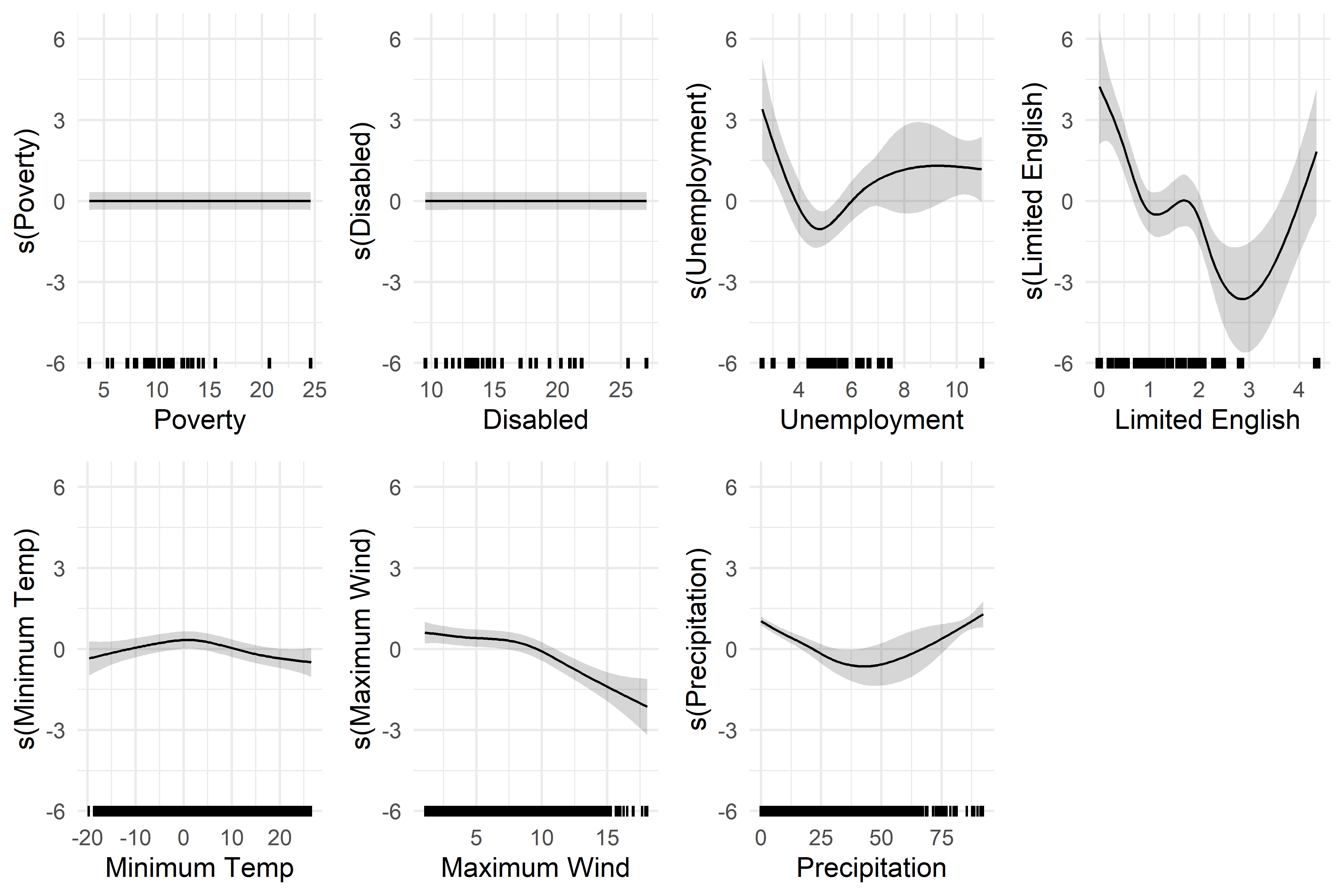

Supplement: S4 Fig — Partial effects from the fitted GAMM model predicting the absence of a power outage for 31 county-utility areas as a function of function of poverty (%), disability (%), square root of the % of limited English, unemployment (%), rural (%), minimum temperature (°C), maximum wind (m/s), and precipitation (mm). The shaded areas represent the 95% confidence intervals for the partial effects, the solid lines represent the smooth fitting curves of outage absence, and the x-axis represent the measured values of the explanatory variables. Rug marks along the x-axis represent data points from the original dataset (n = 39,847) to indicate the distribution of observations. (TIF) [file pone.0307742.s004.tif]

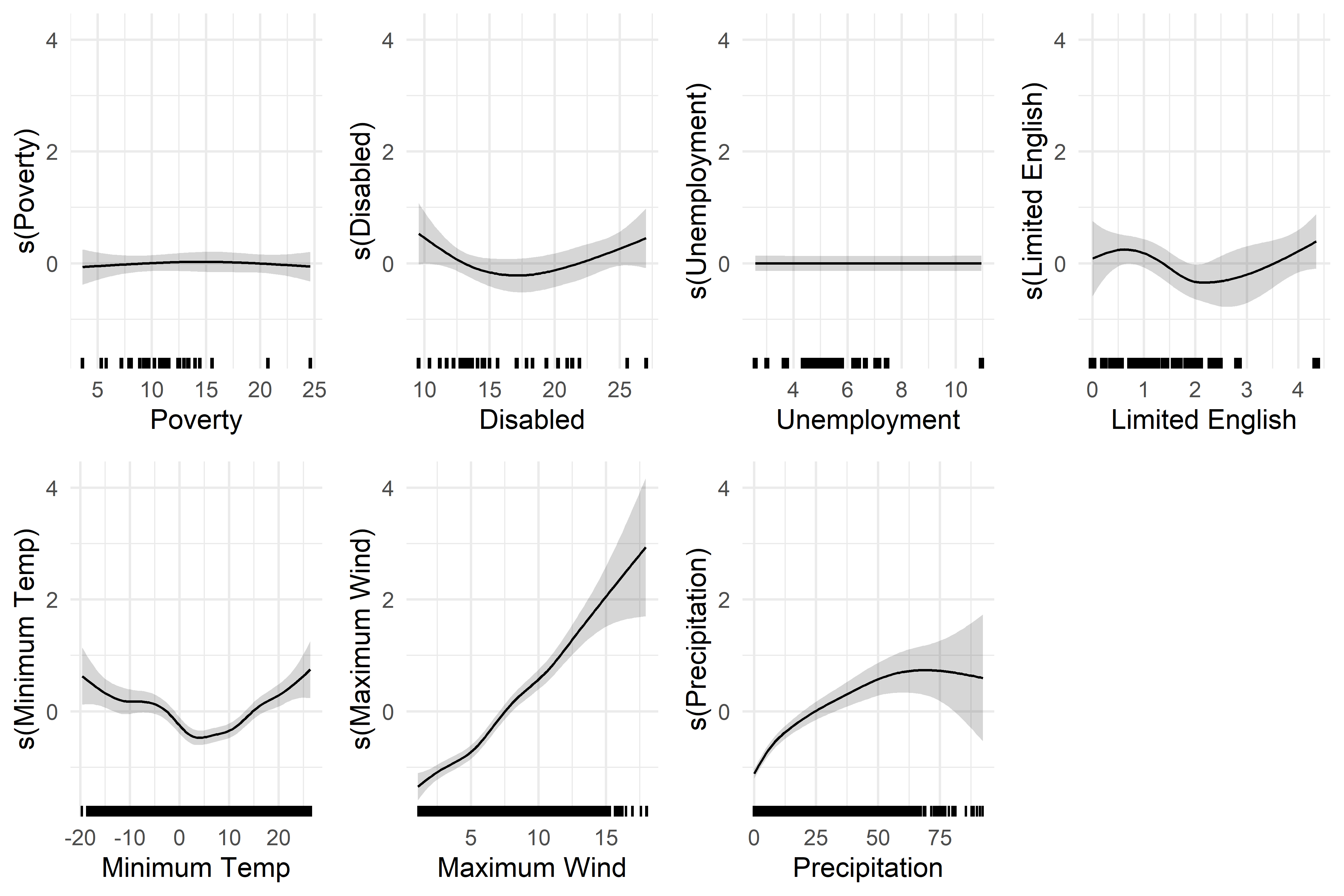

Supplement: S5 Fig — Partial effects from the fitted GAMM predicting daily mean log-transformed SAIDI in Washington counties as a function of poverty (%), disability (%), square root of the % of limited English, unemployment (%), rural (%), minimum temperature (°C), maximum wind (m/s), and precipitation (mm). The shaded areas represent the 95% confidence intervals for the partial effects, the solid lines represent the smooth fitting curves of log(SAIDI) and the x-axis represent the measured values of the explanatory variables. Rug marks along the x-axis represent data points from the original dataset (n = 31,140) to indicate the distribution of observations. (TIF) [file pone.0307742.s005.tif]
