# Supplementary material for: Association of social vulnerability factors with power outage burden in Washington state: 2018–2021
Source: PLoS One. 2024 Sep 4;19(9):e0307742. doi: 10.1371/journal.pone.0307742 (PMC11373849; doi:10.1371/journal.pone.0307742)

Fig S2, Event 1: 2018

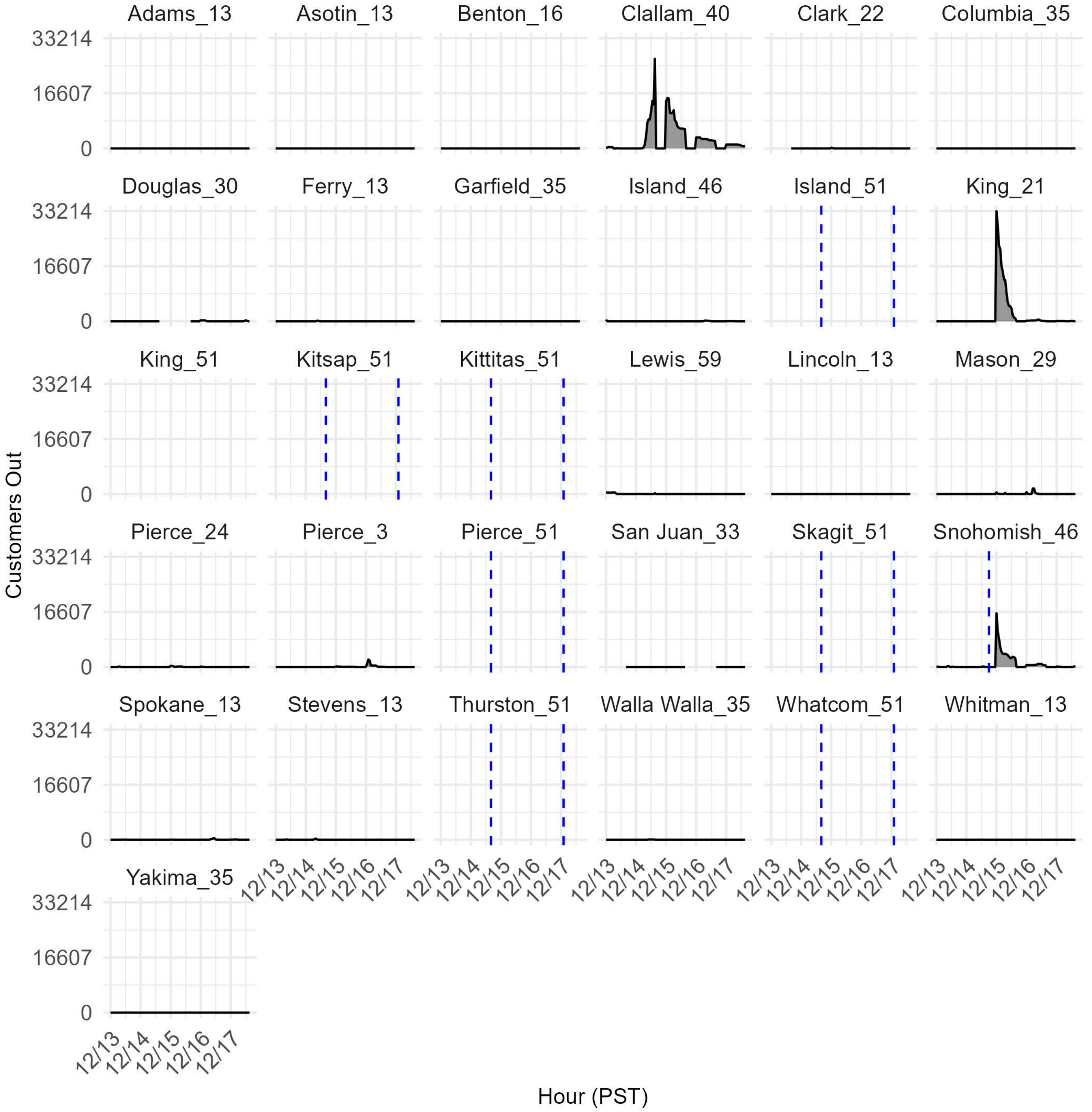

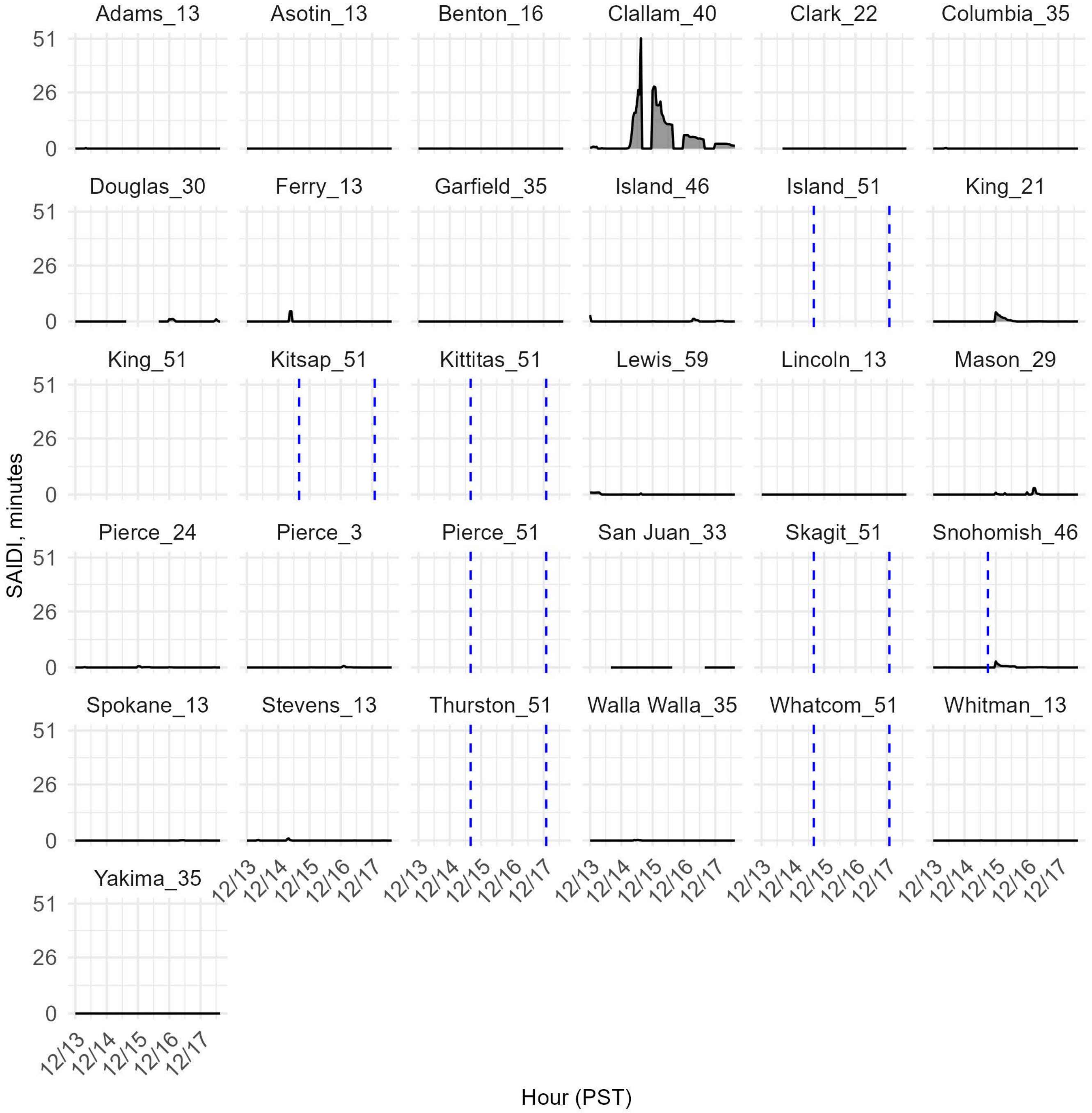

Fig S2, Event 2: 2018

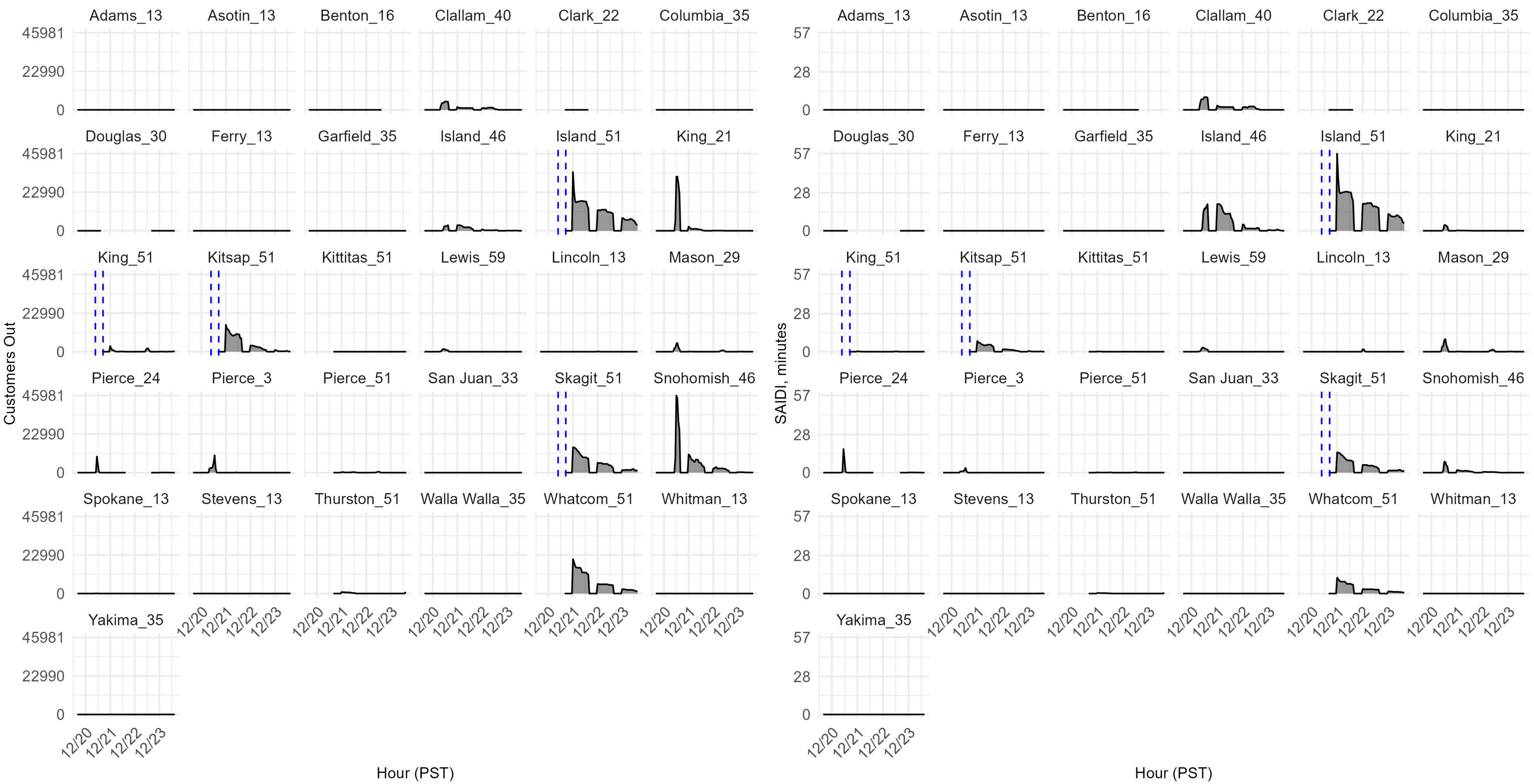

Fig S2, Event 3: 2019

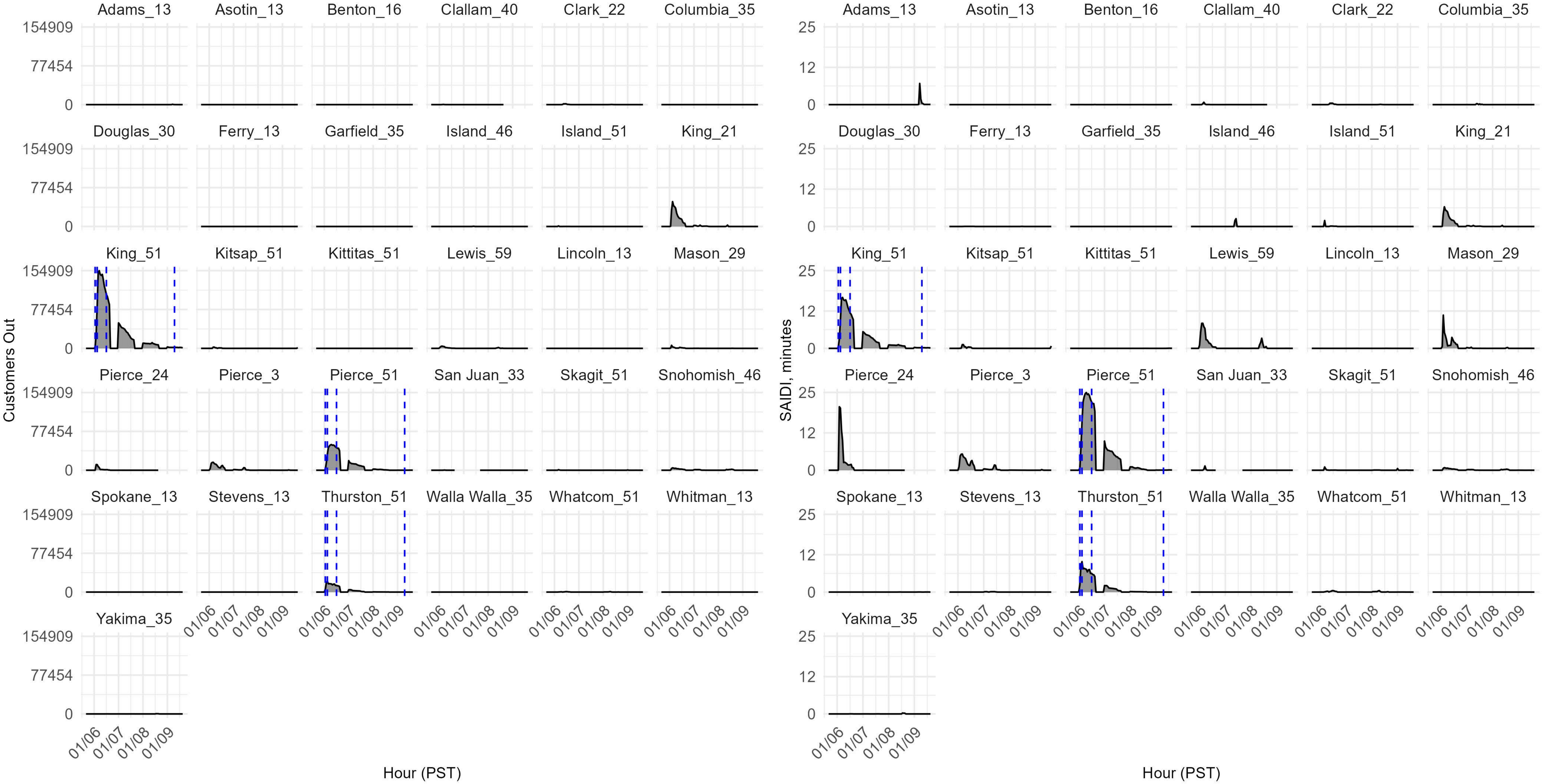

Fig S2, Event 4: 2019

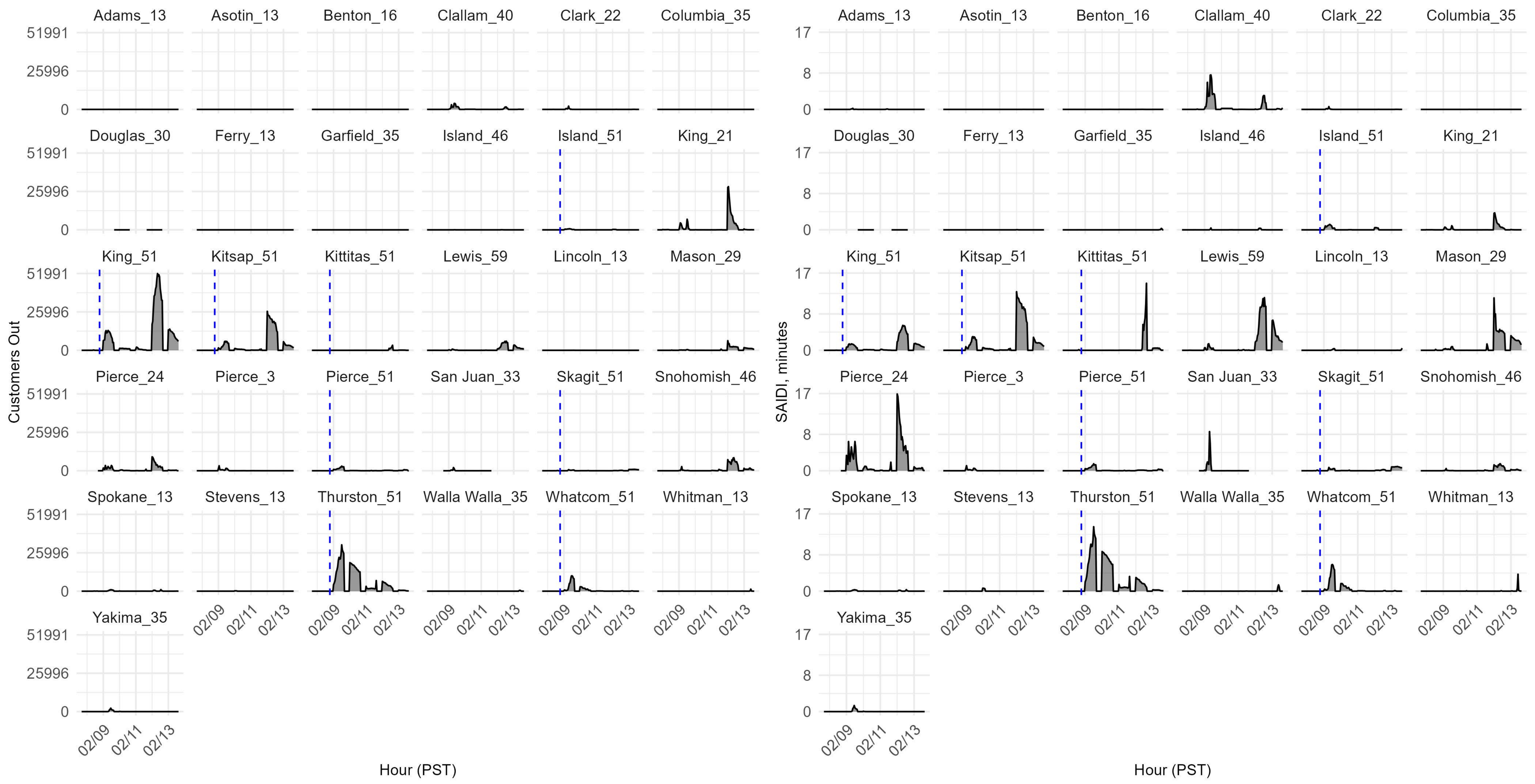

Fig S2, Event 5: 2020

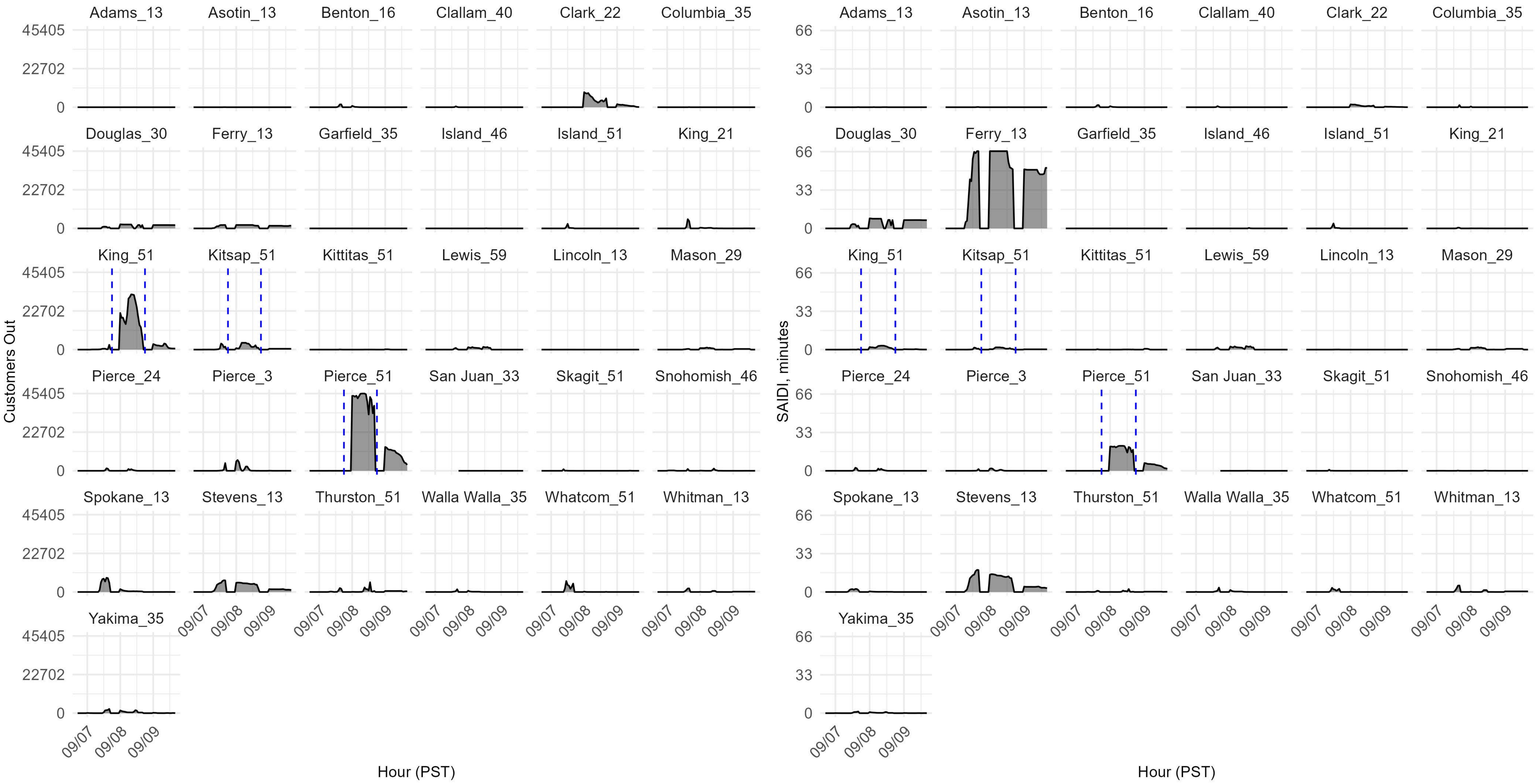

Fig S2, Event 6: 2020

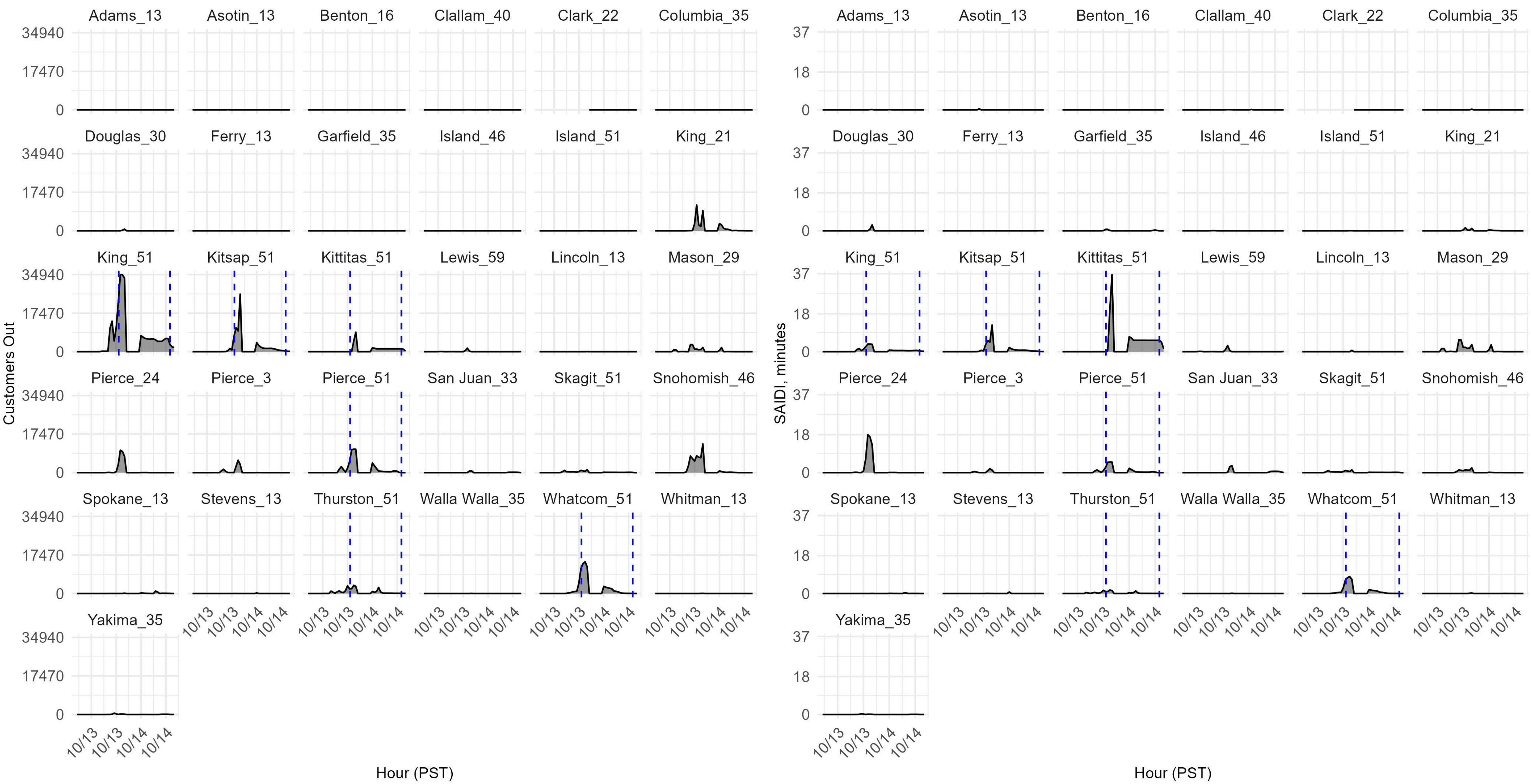

Fig S2, Event 7: 2021

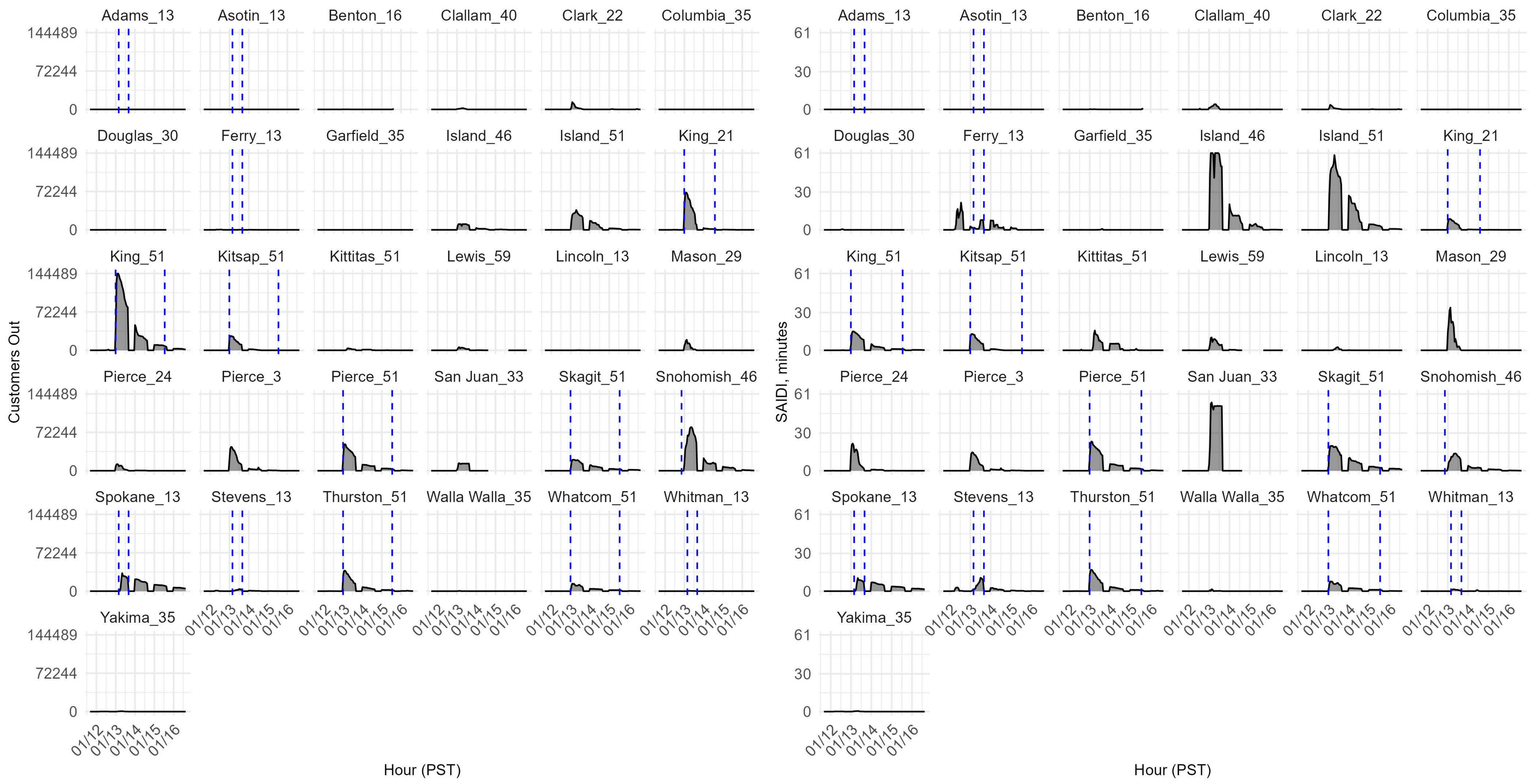

Fig S2, Event 8: 2021

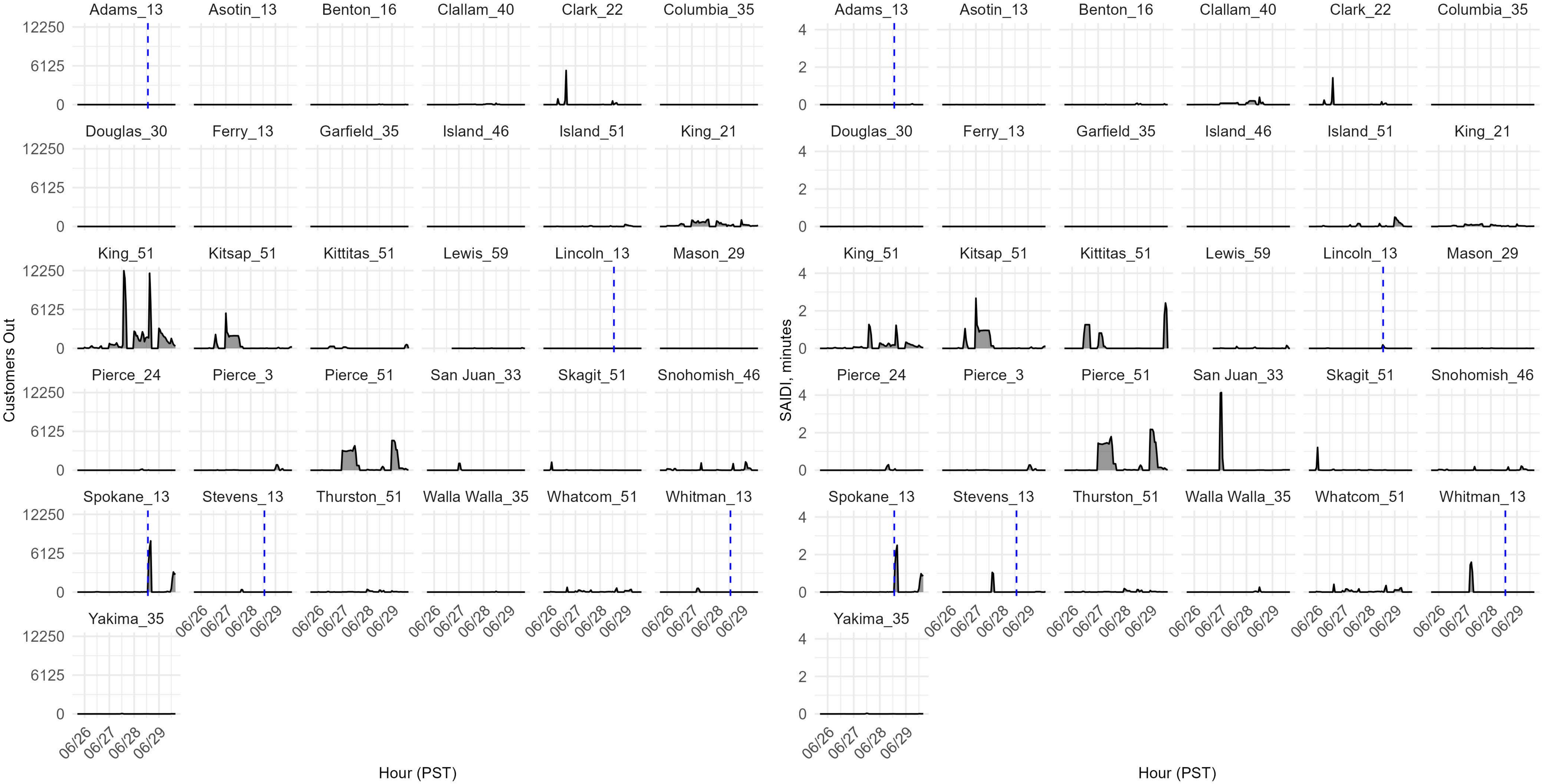

Fig S2, Event 9: 2021

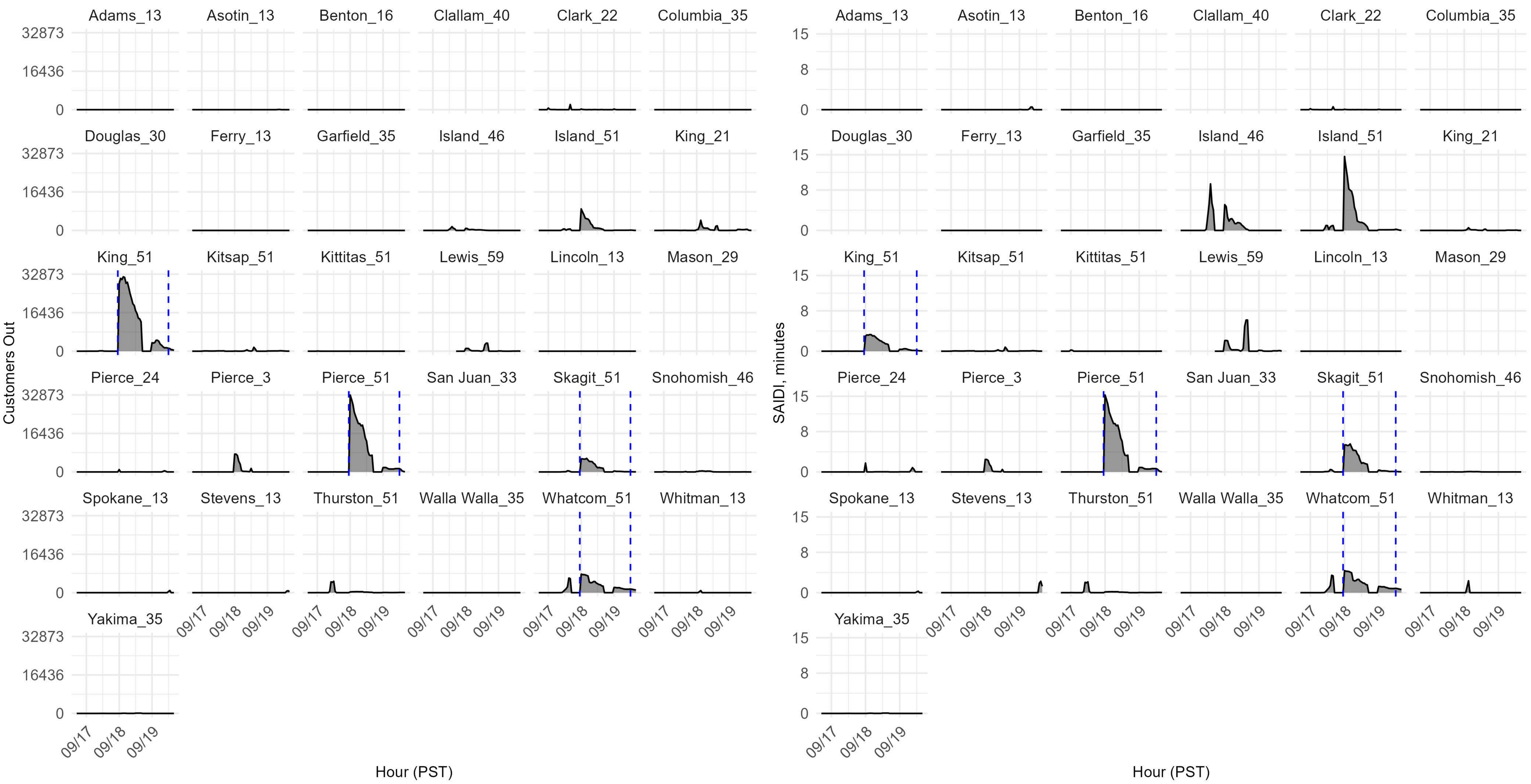

Fig S2, Event 10: 2021

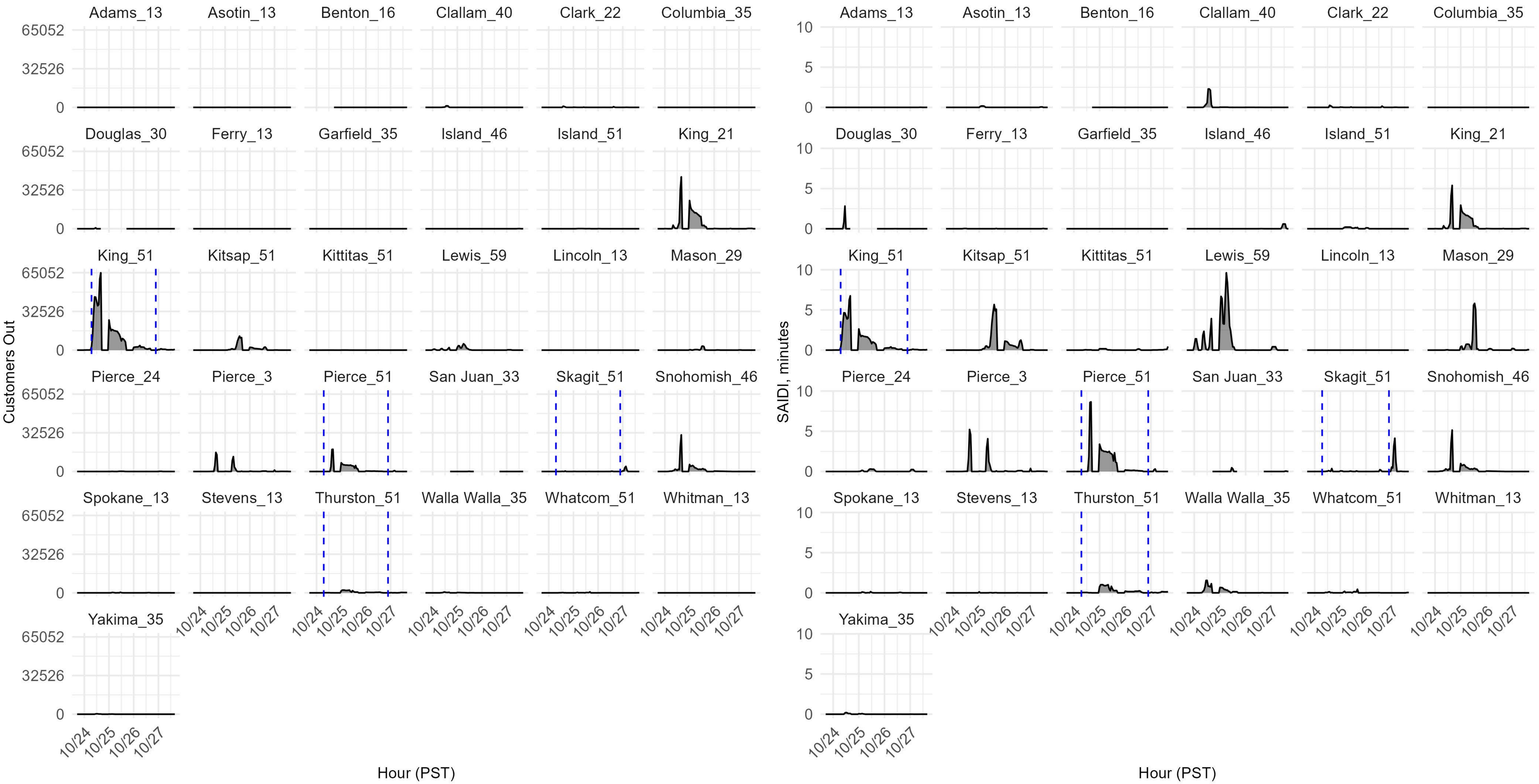

Fig S2, Event 11: 2021

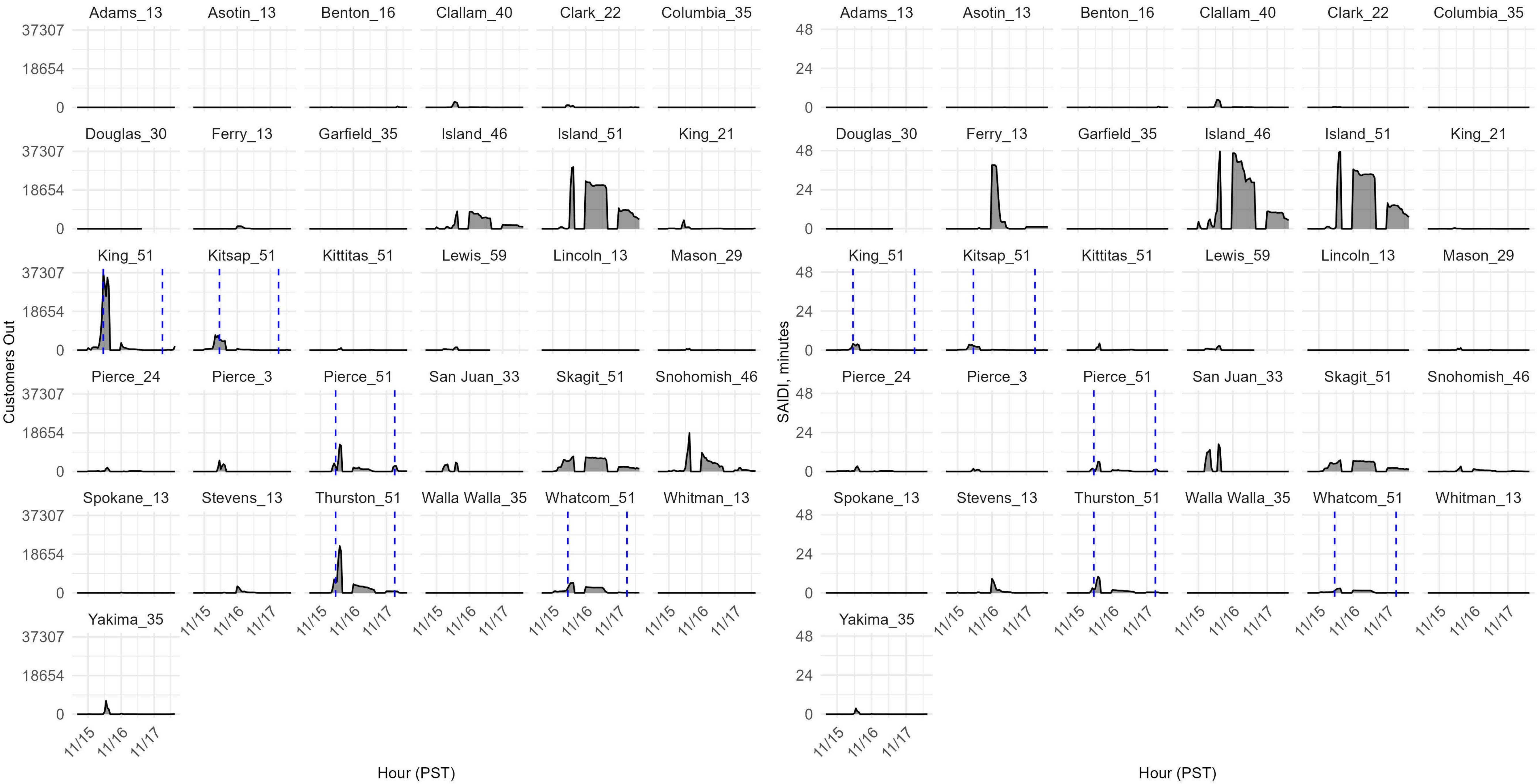

Supplement: S2 Fig — Validation of PowerOutage.US data with the Department of Energy (DOE)-417, “Electric Emergency Incident and Disturbance Report.” Outage events with dashed lines representing major events in the on the DOE-417, “Electric Emergency Incident and Disturbance Report.” [1] Data representing the start and end date and time for the event according to the DOE files is demarcated with a blue dashed line. Areas with a blank x-axis indicate missing PowerOutage.US data. When impacted counties are missing from the DOE data, we assumed all counties in the utility service territory were affected. (PDF) [file pone.0307742.s002.pdf]
