# Supplementary material for: Association of social vulnerability factors with power outage burden in Washington state: 2018–2021
Source: PLoS One. 2024 Sep 4;19(9):e0307742. doi: 10.1371/journal.pone.0307742 (PMC11373849; doi:10.1371/journal.pone.0307742)
Supplement: S1 File — (DOCX) [file pone.0307742.s006.docx]

**S1 File. Data Quality and Supplementary References**

Electric utilities with only partial data and/or low data quality were excluded (S1 and S2 Tables). We excluded four utilities that had no observations until the fall of 2020, three utilities that had frequent gaps in observations of ≥ 200 days, one utility that misassigned POs from one county to another and truncated the number of customers affected. We also excluded observations with unknown county names and county-utility service territories with fewer than 350 customers due to the sparseness of data. Additional observations were removed from primary and secondary analyses due to data fluctuations.

Data reliability and zero values

During the study period, POs were reported on 46.6% to 100% of utility-days. The absence of outage observations raised questions about the underlying reasons. PowerOutage.us documentation suggests that the absence of outage observations means that there were no outages but there were indications that the API was sometimes offline or not reporting outages for an unknown reason. We noted instances where large utilities, which usually log changes via the API daily, showed no recorded data on some days. This pattern was indicative of potential lapses in reporting or API communication issues. For example, a major utility failed to report any POs or changes from October 19 to December 18, 2018, despite a known significant outage event within that period.

Handling missing data

We assumed that the API was offline (missing at random) whenever there was an absence of observations for an entire utility on a calendar day in the raw data. Consequently, we dropped a total of 808 (2.5%) and 3,968 (9.1%) county-utility-days without outage observations for the primary and secondary analyses, respectively. We compiled the PO data by county-utility rather than county because the PowerOutage.us database only included 22% of all utilities in the state and to minimize the number of study days that would be dropped due to missing data.

**Supplementary References**

1. U.S. Energy Information Administration. Major disturbances and unusual occurrences archive [Internet]. Washington, D.C.: U.S. Energy Information Administration; 2022 [cited 2022 Nov 23]. Available from: https://www.eia.gov/electricity/data/disturbance/disturb_events_archive.html

2. Robinson J. Personal Communication. 2022.

3. KOMO Staff. Power outages sweeping Puget Sound as storm hits again. KOMO News [Internet]. 2019 Feb 11 [cited 2022 Nov 23]; Available from: https://komonews.com/news/local/power-outages-sweeping-puget-sound-as-storm-hits-again
