# Supplementary material for: Association of social vulnerability factors with power outage burden in Washington state: 2018–2021
Source: PLoS One. 2024 Sep 4;19(9):e0307742. doi: 10.1371/journal.pone.0307742 (PMC11373849; doi:10.1371/journal.pone.0307742)
Supplement: S1 Table — US data and Issues with Zero Values. (DOCX) [file pone.0307742.s007.docx]

**S1 Table. Example of PowerOutage.US data and Issues with Zero Values**

| **Utility** | **State** | **County** | **Subdivision** | **CustomersOut** | **RecordedDateTime** | **Zero Values** |
| --- | --- | --- | --- | --- | --- | --- |
| Puget Sound Energy | WA | Pierce | Puyallup | 111 | 2018-09-08 04:26:24 | A zero value at a different date time often indicates the end of an outage. |
| Puget Sound Energy | WA | Pierce | Puyallup | 68 | 2018-09-08 05:57:02 |  |
| Puget Sound Energy | WA | Pierce | Puyallup | 62 | 2018-09-08 14:25:46 |  |
| Puget Sound Energy | WA | Pierce | Puyallup | 0 | 2018-09-08 14:56:08 |  |
| Puget Sound Energy | WA | Pierce | Puyallup | 2 | 2018-09-10 16:28:00 |  |
| Puget Sound Energy | WA | Island | Bells Beach | 746 | 2021-11-17 17:59:45 | A different date-time stamp of zero indicates a problem receiving data [2]. |
| Puget Sound Energy | WA | Island | Bells Beach | 0 | 2021-11-17 18:20:14 |  |
| Puget Sound Energy | WA | Island | Bells Beach | 746 | 2021-11-17 19:18:54 |  |
| Puget Sound Energy | WA | Island | Bells Beach | 0 | 2021-11-17 20:03:19 |  |
| Puget Sound Energy | WA | Island | Bells Beach | 740 | 2021-11-17 20:42:26 |  |
| Puget Sound Energy | WA | Island | Coupeville | 2765 | 2021-12-22 14:38:15 | This pattern indicates that there was an error checking for data and by the time the error was corrected, the outage was fixed [2]. |
| Puget Sound Energy | WA | Island | Coupeville | 2764 | 2021-12-22 15:08:27 |  |
| Puget Sound Energy | WA | Island | Coupeville | 0 | 2021-12-22 15:08:27 |  |
| Puget Sound Energy | WA | Island | Coupeville | 6 | 2021-12-22 15:48:27 |  |
