# Supplementary material for: Association of social vulnerability factors with power outage burden in Washington state: 2018–2021
Source: PLoS One. 2024 Sep 4;19(9):e0307742. doi: 10.1371/journal.pone.0307742 (PMC11373849; doi:10.1371/journal.pone.0307742)
Supplement: S2 Table — (DOCX) [file pone.0307742.s008.docx]

S2 Table. Data Processing

| **Category** | **Description** | **Rationale** |
| --- | --- | --- |
| County Name Substitution | For observations with unknown or incorrect county names, substitutions were made based on various conditions, when possible (*N*=2,315 [0.57%], *N*=3,507 [0.70%], for primary and secondary analysis, respectively). Otherwise, observations were dropped. | Single county: Substituted with the only county served by utility. Multiple counties: Used relationships between subdivisions and counties within PowerOutage.us and maps. |
| Customer-Outages | For a utility found to report all customer-outages smaller than 5 as 4, values of 4 were substituted with 1. | "1" is the most frequent non-zero integer for customer-outages, comprising 54.9% of all values between 1 and 4. |
| API Responsiveness | We ended all outages with duplicate date-time stamp and non-zero observations. | The API may be considered unresponsive when there is a duplicated county-utility-subdivision-date-time and zero outage value. However, it is unknown whether these patterns are API errors or the end of the outage. |
| Outlier Exclusion Strategy | Removed zero values within patterns of N, 0, N for cases where customers out exceeded 20 on both sides of the zero and if non-zero values occurred within 2 hours (*n =* 1,406 [0.34%], *n =* 5,652 [1.1%], for primary and secondary analysis respectively.) | Small outages are common, large outages are rare.  Large values of N, patterns of N, 0, N suggest problems with utility reporting |
|  | We removed the first zero of N, 0, N, 0 patterns when the pattern occurred within 1 hour (N=981, [0.24%] and 2,609 [0.5%], for primary and secondary analysis respectively.) | Patterns of N, 0, N, 0 occurring within a short duration indicate issues with the API reporting outages, even for small N. |
| Missing Outage End | We ended all outages when there was a non-zero outage observation without any other reporting of outages within 96 hours (*n =* 251 [0.027%].) | Outages are unlikely to remain unchanged for four days and this pattern likely represents an API error. |
| Misclassification | We reclassified the county name for 37 observations for one county-utility area. | The number of customers affected greatly exceeded the customers served in the county. Per communication with PowerOutage.us, counties were misclassified from the larger county. This may occur for utilities that only provide Geo data like a point, close to a county border. The outages are assigned to the county at that point, even though the outage itself could affect an area that crosses town/county borders [2]. |
