# Supplementary material for: Association of social vulnerability factors with power outage burden in Washington state: 2018–2021
Source: PLoS One. 2024 Sep 4;19(9):e0307742. doi: 10.1371/journal.pone.0307742 (PMC11373849; doi:10.1371/journal.pone.0307742)
Supplement: S3 Table — aStudy data for 2018 was only a partial year and is not presented. bStatewide study data includes 15 utilities, while EIA data consists of all reporting utilities statewide. The EIA SAIDI values include major events from the EIA Electric Annual Power Report for Washington State. Utilities shaded in gray are included in the primary analysis, utilities shaded in white are additionally included in the secondary analysis. Empty rows indicate missing EIA data. (DOCX) [file pone.0307742.s009.docx]

**S3 Table. Annual System Average Interruption Duration Index (SAIDI) for Individual Utilities and State: Study vs. EIA Estimates from 2019-2021**

|  | **2019**^a^ | | **2020** | | **2021** | |
| --- | --- | --- | --- | --- | --- | --- |
|  | **Study Estimate** | **EIA Data** | **Study Estimate** | **EIA Data** | **Study Estimate** | **EIA Data** |
| **Statewide**^b^ | 270 | 311 | 234 | 269 | 476 | 555 |
| **3** | 124 | 125 | 88 | 85 | 322 | 314 |
| **13** | 238 | 222 | 327 | 218 | 485 | 548 |
| **16** | 63 | 44 | 90 | 89 | 131 | 60 |
| **21** | 192 | 179 | 93 | 80 | 284 | 245 |
| **22** | 49 | 98 | 90 | 121 | 98 | 122 |
| **24** | 757 | 161 | 262 | 77 | 463 | 52 |
| **29** | 386 | 638 | 325 | 452 | 552 | 419 |
| **30** | 149 |  | 661 |  | 147 |  |
| **33** | 438 | 325 | 211 | 222 | 1288 |  |
| **35** | 131 | 130 | 304 | 286 | 145 | 135 |
| **40** | 264 | 162 | 687 | 250 | 736 | 561 |
| **46** | 113 | 125 | 229 | 256 | 583 | 643 |
| **51** | 536 | 550 | 404 | 414 | 864 | 849 |
| **59** | 899 | 881 | 342 | 433 | 737 | 897 |

^a^Study data for 2018 was only a partial year and is not presented. ^b^Statewide study data includes 15 utilities, while EIA data consists of all reporting utilities statewide. The EIA SAIDI values include major events from the EIA Electric Annual Power Report for Washington State. Utilities shaded in gray are included in the primary analysis, utilities shaded in white are additionally included in the secondary analysis. Empty rows indicate missing EIA data.
