# Supplementary material for: Association of social vulnerability factors with power outage burden in Washington state: 2018–2021
Source: PLoS One. 2024 Sep 4;19(9):e0307742. doi: 10.1371/journal.pone.0307742 (PMC11373849; doi:10.1371/journal.pone.0307742)
Supplement: S4 Table — (DOCX) [file pone.0307742.s010.docx]

**S4 Table. Major Events Reported to the Department of Energy (DOE) on the OE-417 “Electric Emergency Incident and Disturbance Report”**

| **Events** | **Start Date-Time** | **End Date-Time** | **Duration** | **Customers Affected** | **Counties** | **Comments** |
| --- | --- | --- | --- | --- | --- | --- |
| Event 1 | 12/14/2018 16:00 | 12/17/2018 2:00 | 58 Hrs, 0 Min | 150,000 | King, Kitsap, Island, Pierce, Thurston, Whatcom, Skagit, Kittitas | Not identified in PowerOutage.us (POUS) data. API was offline for utility 51. |
|  | 12/14/2018 18:00 | Unknown | Unknown | 60,000 | Snohomish | Utility 46 |
| Event 2 | 12/20/2018 9:30 | 12/20/2018 17:00 | 7 Hrs, 30 Min | 165,000 | Skagit, Snohomish, King, Kitsap, Island | API for utility 51 was offline prior to outage started |
| Event 3 | 1/6/2019 1:00 | 1/6/2019 12:00 | Unknown | 230,000 | King, Thurston, Pierce | Utility 51 |
|  | 1/6/2019 3:00 | 1/9/2019 7:00 | 76 Hrs, 0 Min | 230,000 | Unknown | Utility 51 |
| Event 4 | 2/8/2019 18:30 | Unknown | Unknown | 50,940 | Unknown | Utility 51  Additional outages were missing from OE-417. A large outage on February 11, 2019 to February 12, 2019 [3] affecting utility #51 noted in the POUS data. |
| Event 5 | 9/7/2020 9:13 | 9/8/2020 6:00 | 20 Hrs, 47 Min | 21,000 | Unknown | Okanogan County not in POUS data. |
|  | 9/7/2020 18:00 | 9/8/2020 18:00 | 24 Hrs, 0 Min | 71,500 | Kitsap, King, Pierce | Utility 51 |
| Event 6 | 10/13/2020 13:14 | 10/14/2020 14:00 | 24 Hrs, 46 Min | 76,000 | Whatcom, King, Pierce, Thurston, Kittitas, Kitsap | Utility 51 |
| Event 7 | 1/12/2021 20:30 | Unknown | Unknown | 62,000 | Snohomish | Utility 46 |
|  | 1/12/2021 23:49 | 1/14/2021 14:00 | 38 Hrs, 11 Min | 70,000 | Unknown, but must be King due to named utility | Utility 21 |
|  | 1/12/2021 23:50 | 1/15/2021 13:00 | 61 Hrs, 10 Min | 320,000 | Kitsap, Thurston, King, Pierce, Skagit, Whatcom | Utility 51 |
|  | 1/13/2021 3:43 | 1/13/2021 16:00 | 12 Hrs, 17 Min | 70,417 | Unknown | DOE data combined Idaho and Washington, while POUS only included Washington. |
| Event 8 | 6/28/2021 13:20 | Unknown | Unknown | Unknown | Spokane, Stevens, Lincoln, Adams, Whitman | Heat dome, only utility #13 listed due to rotating blackouts. |
| Event 9 | 9/17/2021 23:00 | 9/19/2021 12:00 | 37 Hrs, 0 Min | 87,000 | Pierce, Whatcom, Skagit, King | Utility 51 |
| Event 10 | 10/24/2021 7:00 | 10/26/2021 21:30 | 62 Hrs, 30 Min | 233,000 | King, Pierce, Skagit, Thurston | Utility 51 |
| Event 11 | 11/15/2021 11:00 | 11/17/2021 6:00 | 43 Hrs, 0 Min | 90,000 | King, Kitsap, Whatcom, Thurston, Pierce | Utility 51 |
