# Supplementary material for: Association of social vulnerability factors with power outage burden in Washington state: 2018–2021
Source: PLoS One. 2024 Sep 4;19(9):e0307742. doi: 10.1371/journal.pone.0307742 (PMC11373849; doi:10.1371/journal.pone.0307742)
Supplement: S5 Table — n = 39,847 County-Utility Days. aOutages of 8 hours or more could start and end on different calendar days; all days are included. bTmed was 22.12 minutes for all 31 county-utility territories. (DOCX) [file pone.0307742.s011.docx]

**S5 Table. Daily SAIDI and Maximum Fraction of Customers Out by Major Event Definitions for the Secondary Analysis**

|  | **Non-Zero Outages^a^** | **≥ 0.1% for 8 Hr^a^** | **Daily SAIDI > T_med_^b^** | **≥ 10,000 Max Affected** | **≥ 50,000 Max Affected** |
| --- | --- | --- | --- | --- | --- |
| **Sample Size, d** | 31,126 | 1,557 | 404 | 161 | 9 |
| **Daily SAIDI, min**  *Median (Q1-Q3)* | 0.06 (0.02, 0.3) | 3.42 (1.38, 10.74) | 50.5 (30.68, 111.82) | 50.35 (17.71, 132.34) | 215.53 (82.45, 235.97) |
| *Range* | 0-1432.03 | 0.09-1432.03 | 22.15-1432.03 | 2.06-1178.31 | 66.64-427.47 |
| **Max Customers Affected**  *Median (Q1-Q3)* | 19.76 (3.62, 115.33) | 335.36 (92, 1262.68) | 3,699.33 (1,318.17, 10,923.38) | 15,940.44 (12,452.02, 26,899.15) | 65,051.62 (51,122.07, 81,959.87) |
| *Range* | 0.01-154,908.77 | 1.19-44,863.97 | 58-154,908.77 | 10,031.6-154,908.77 | 50,074.21-154,908.77 |
| **Max Fraction of Customers Affected**  *Median (Q1-Q3)* | 0.003 (0.0001 0.0015) | 0.012 (0.004, 0.036) | 0.13 (0.08, 0.27) | 0.11 (0.06, 0.28) | 0.23 (0.11, 0.27) |
| *Range* | 0-1 | 0.001-1 | 0.02-1 | 0.02-1 | 0.09-0.42 |
| **Customer-Hr (Thousands)**  *Median (Q1-Q3)* | 0.07 (0.01, 0.44) | 1.81 (0.49, 6.15) | 26.49 (7.68, 92.52) | 133.7 (54.88, 261.33) | 782.01 (769.5, 1286.36) |
| *Range* | 0-2272.32 | 0-596.42 | 0.47-2272.32 | 13.22-2272.32 | 630.51-2272.32 |

*n =* 39,847 County-Utility Days. ^a^Outages of 8 hours or more could start and end on different calendar days; all days are included. ^b^T_med_ was 22.12 minutes for all 31 county-utility territories.
