# Supplementary material for: Association of social vulnerability factors with power outage burden in Washington state: 2018–2021
Source: PLoS One. 2024 Sep 4;19(9):e0307742. doi: 10.1371/journal.pone.0307742 (PMC11373849; doi:10.1371/journal.pone.0307742)
Supplement: S6 Table — Shaded cells are those variables included in analyses. aPercent of Medicare Population; bSquare root transformed. Poverty is defined as less than 100% of the federal poverty limit. BIPOC: Black Indigenous or Person of Color. DME: Electricity Dependent Durable Medical Equipment, Unemp: Unemployed civilian population. (DOCX) [file pone.0307742.s012.docx]

**S6 Table. Pearson’s Correlation for Social Vulnerability Factors (*n =* 31 County-Utility Areas, Secondary Analysis)**

|  | Disability,% | Unemp, % | Poverty, % | Sqrt of % Limited English | Multiunit Housing, % | DME, %^a^ | Age ≥ 65 yrs alone, % | Age ≤ 5 yrs, % | BIPOC, % | Mobile Home, % | Rural, % | Log(Household Density) |
| --- | --- | --- | --- | --- | --- | --- | --- | --- | --- | --- | --- | --- |
| Disability, % | 1 |  |  |  |  |  |  |  |  |  |  |  |
| Unemp, % | 0.35*** | 1 |  |  |  |  |  |  |  |  |  |  |
| Poverty, % | -0.01 | 0.42*** | 1 |  |  |  |  |  |  |  |  |  |
| Sqrt of % Limited English | -0.53*** | -0.03*** | 0.31*** | 1 |  |  |  |  |  |  |  |  |
| Multiunit Housing, % | -0.69*** | -0.07*** | 0.20*** | 0.46*** | 1 |  |  |  |  |  |  |  |
| DME, %^a^ | 0.61*** | 0.38*** | 0.56*** | -0.20*** | -0.30*** | 1 |  |  |  |  |  |  |
| Age ≥ 65 yrs alone, % | 0.79*** | 0.20*** | -0.09*** | -0.65*** | -0.59*** | 0.41*** | 1 |  |  |  |  |  |
| Age ≤ 5 yrs, % | -0.24*** | 0.01* | 0.11*** | 0.70*** | 0.07*** | -0.04*** | -0.56*** | 1 |  |  |  |  |
| BIPOC, % | -0.38*** | 0.03*** | 0.27*** | 0.91*** | 0.36*** | -0.10*** | -0.56*** | 0.75*** | 1 |  |  |  |
| Mobile Home, % | 0.67*** | 0.46*** | 0.40*** | -0.08*** | -0.66*** | 0.61*** | 0.38*** | 0.20*** | 0.04*** | 1 |  |  |
| Rural, % | 0.69*** | 0.24*** | 0.20*** | -0.46*** | -0.68*** | 0.54*** | 0.70*** | -0.40*** | -0.40*** | 0.69*** | 1 |  |
| Log(Household Density) | -0.67*** | -0.32*** | -0.51*** | 0.24*** | 0.57*** | -0.80*** | -0.52*** | 0.13*** | 0.21*** | -0.78*** | -0.79*** | 1 |

Shaded cells are those variables included in analyses.

^a^Percent of Medicare Population; ^b^Square root transformed.

Poverty is defined as less than 100% of the federal poverty limit. BIPOC: Black Indigenous or Person of Color. DME: Electricity Dependent Durable Medical Equipment, Unemp: Unemployed civilian population.
