# Supplementary material for: Association of social vulnerability factors with power outage burden in Washington state: 2018–2021
Source: PLoS One. 2024 Sep 4;19(9):e0307742. doi: 10.1371/journal.pone.0307742 (PMC11373849; doi:10.1371/journal.pone.0307742)
Supplement: S7 Table — edf: effective degrees of freedom. Missing data: n = 3,968 (9.1%) county-utility days. For brevity, we exclude the individual JDay and DayInYear smooths for each county-utility. aYear reference category is 2018 for the Gaussian model and models including JDay do not include a categorical variable for Year. bIndicator variable for limited English is transformed by taking the square root of its values. cThe variable to capture temporality and seasonality is JDay for the binomial model and DayInYear for the Gaussian model. dThe best fit model for the absence/presence model in the secondary analysis included a global term for seasonality (JDay). (DOCX) [file pone.0307742.s013.docx]

**S7 Table. Summary Table of Generalized Additive Mixed Model (GAMM) for ZALN Model of SAIDI (Secondary Analysis)**

|  | **Secondary Analysis** | | | |
| --- | --- | --- | --- | --- |
|  | **Binomial (Absence of Outage)** | | **Gaussian** | |
| **Parametric Coefficients** | | | | |
| Component | Estimate | P-value | Estimate | P-value |
| Intercept | -4.04 | <0.001 | -0.54 | <.0001 |
| Year^a^ |  | | | |
| 2019 |  | | -0.16 | <.0001 |
| 2020 |  |  | -0.15 | <.0001 |
| 2021 |  |  | -0.04 | 0.341 |
| **Approximate Significance of Smooth Terms** | | | | |
| Component | edf | P-value | edf | P-value |
| s(Poverty) | 0.00 | 0.354 | 0.21 | 0.232 |
| s(Disability) | 0.00 | 0.364 | 1.30 | 0.018 |
| s(Unemployment) | 2.71 | 0.004 | 0.00 | 0.559 |
| s(Square Root of Limited English)^b^ | 4.44 | 0.004 | 1.83 | 0.009 |
| s(Minimum Temperature) | 4.11 | <.0001 | 6.03 | <.0001 |
| s(Max Wind Speed) | 4.14 | <.0001 | 4.96 | <.0001 |
| s(Precipitation) | 2.45 | <.0001 | 4.30 | <.0001 |
| s(DayInYear): Year^c^ |  | | | |
| 2018 |  | | 6.51 | <.0001 |
| 2019 |  |  | 4.28 | <.0001 |
| 2020 |  |  | 7.09 | <.0001 |
| 2021 |  |  | 6.86 | <.0001 |
| JDay^d^ | 0.00 | 0.961 |  | |
| s(countyID) | 21.83 | <.0001 | 26.25 | <.0001 |
| **Model Fit** | | | | |
|  | **Binomial** | | **Gaussian** | |
| Deviance explained | 0.44 | | 0.17 | |
| N | 39,847 | | 31,126 | |

edf: effective degrees of freedom. Missing data: *n* = 3,968 (9.1%) county-utility days. For brevity, we exclude the individual JDay and DayInYear smooths for each county-utility. ^a^Year reference category is 2018 for the Gaussian model and models including JDay do not include a categorical variable for Year.^b^Indicator variable for limited English is transformed by taking the square root of its values. ^c^The variable to capture temporality and seasonality is JDay for the binomial model and DayInYear for the Gaussian model. ^d^The best fit model for the absence/presence model in the secondary analysis included a global term for seasonality (JDay).
