# Supplementary material for: Association of social vulnerability factors with power outage burden in Washington state: 2018–2021
Source: PLoS One. 2024 Sep 4;19(9):e0307742. doi: 10.1371/journal.pone.0307742 (PMC11373849; doi:10.1371/journal.pone.0307742)
Supplement: Attachment — Submitted filename: review0521.docx [file pone.0307742.s014.docx]

This paper studies the relationships between power outages and social economic status of communities using data from Washington state. The paper consists of three aspects. First, the authors conduct data processing, with several more careful treatments that were not done by the prior work. Second, the paper proposes a model to relate power outages with social economic status of communities, i.e., Social Vulnerability Indices from CDC. Third, the paper uses the data to fit model parameters, and analyzes the results.

The strengths of the paper include careful and meticulous data processing. In particular, the authors separate major events from moderate disruptions using IEEE standards, and removing spurious data samples. Such efforts set the stage for data analysis. The model used appears to be different from those of the prior work. Overall, the study is relevant as Washington state suffers from frequent weather-induced power outages. Such a study has not been done in the prior work.

Weaknesses of the paper and suggestions include the following.

-First of all, the paper needs a major rewrite to be accessible by readers. For example, the paper can be structured to keep the key aspects of the study in the main text while moving the detailed implementation to the supplementary sections. For instance, the data section may keep what is needed for preprocessing and why that is important. How to do processing can be in the supplementary.

-The model equations, e.g., Equ (3) and (5) need to use standard and clear mathematical expressions. For example, what is the ``dependency” terms? Are covariate weighted linearly? Also, how to use the model also needs to be explained: are model parameters the same for all counties? Or the Bernoulli process pointwise, i.e., with different parameters for different counties? How are the model parameters estimated? Software used to implement the model can be moved to the supplementary.

-Analysis of the results is now in ``discussion” section, which seems to be too long to be clear. To highlight the findings, the results and analysis can be a separate section. It needs to be made clear what key results are obtained, and how the results differ from the prior work (and why if known). For example, in what way, the outage durations are non-linear in SVI? Also, which part of the extensive data processing makes the difference to the results? Why are the impact of outages inconsistent in terms of SVIs?

Some detailed comments:

-It will be more clear to specify the actual vertical axis in Figs 3, 4, 5, 6 (rather than ``partial effects”).

-The paper uses DoE criterion for major events, i.e., outages that affected more than 50,000 (or >10,000) customers. Such a criterion is for transmission grid. For distribution grid, separating major events from moderate ones are considered in a prior work below.

Reference: Afsharinejad, A., et al., “Large-scale data analytics for resilient recovery services from power failures,” Joule Cell Press, 2021

Overall, this reviewer is positive about the paper but thought a revision is needed.
