# Supplementary material for: Association of social vulnerability factors with power outage burden in Washington state: 2018–2021
Source: PLoS One. 2024 Sep 4;19(9):e0307742. doi: 10.1371/journal.pone.0307742 (PMC11373849; doi:10.1371/journal.pone.0307742)
Supplement: Attachment — Submitted filename: Response to reviewers.docx [file pone.0307742.s015.docx]

The Editor, Reviewers,

PLOS ONE

Date: June 15, 2024

Subject: Addressing Editor/Reviewer Comments for PONE-D-24-00148

Dear Editor and Reviewers,

Thank you for your helpful feedback. We have tried to address your concerns to the best of our abilities. We believe these revisions greatly strengthen the paper. In the table below, we provide our responses to the comments and actions that we took to address them. All changes are highlighted in the marked-up copy.

Best,

The Authors of PONE-D-24-00148

| **Editor** | |
| --- | --- |
| **Comment** | **Action(s) performed** |
| 1. Please ensure that your manuscript meets PLOS ONE's style requirements, including those for file naming. The PLOS ONE style templates can be found at  [https://journals.plos.org/plosone/s/file?id=wjVg/PLOSOne_formatting_sample_main_body.pdf](https://nam12.safelinks.protection.outlook.com/?url=https%3A%2F%2Furldefense.com%2Fv3%2F__https%3A%2F%2Fjournals.plos.org%2Fplosone%2Fs%2Ffile%3Fid%3DwjVg*PLOSOne_formatting_sample_main_body.pdf__%3BLw!!JmPEgBY0HMszNaDT!sArbiVI8lcMDYKo3S1KpXBoOGPUsT9WrXZWzubyjRnOz-kbbJeTTkM0B5uiprKanagtvyIm-V_V53fFchWNo%24&data=05%7C02%7Cclaire.richards%40wsu.edu%7C22187065e67f4fb841b208dc7b415568%7Cb52be471f7f147b4a8790c799bb53db5%7C0%7C0%7C638520768072026803%7CUnknown%7CTWFpbGZsb3d8eyJWIjoiMC4wLjAwMDAiLCJQIjoiV2luMzIiLCJBTiI6Ik1haWwiLCJXVCI6Mn0%3D%7C80000%7C%7C%7C&sdata=kL7a0vrlUaT%2BwRxYyjsUKFk06buto1USANslLkumEac%3D&reserved=0) and  [https://journals.plos.org/plosone/s/file?id=ba62/PLOSOne_formatting_sample_title_authors_affiliations.pdf](https://nam12.safelinks.protection.outlook.com/?url=https%3A%2F%2Furldefense.com%2Fv3%2F__https%3A%2F%2Fjournals.plos.org%2Fplosone%2Fs%2Ffile%3Fid%3Dba62*PLOSOne_formatting_sample_title_authors_affiliations.pdf__%3BLw!!JmPEgBY0HMszNaDT!sArbiVI8lcMDYKo3S1KpXBoOGPUsT9WrXZWzubyjRnOz-kbbJeTTkM0B5uiprKanagtvyIm-V_V53enyKrWR%24&data=05%7C02%7Cclaire.richards%40wsu.edu%7C22187065e67f4fb841b208dc7b415568%7Cb52be471f7f147b4a8790c799bb53db5%7C0%7C0%7C638520768072032176%7CUnknown%7CTWFpbGZsb3d8eyJWIjoiMC4wLjAwMDAiLCJQIjoiV2luMzIiLCJBTiI6Ik1haWwiLCJXVCI6Mn0%3D%7C80000%7C%7C%7C&sdata=hrpC7f5qVykX%2BjAOdcJ2hdpbs8ESB7mZO3mOEaRRidM%3D&reserved=0) | Thank you for the opportunity to correct the style. We corrected the references to fit the PLOS ONE style and a few errors in the style. We rounded numbers in Table 1 for ease of reading. Additionally, we uploaded all supplementary tables as individual files. |
| 2. Thank you for stating the following financial disclosure: "Washington State University New Faculty Seed Grant [PG00019865]." Please state what role the funders took in the study.  If the funders had no role, please state: ""The funders had no role in study design, data collection and analysis, decision to publish, or preparation of the manuscript.""  If this statement is not correct you must amend it as needed.  Please include this amended Role of Funder statement in your cover letter; we will change the online submission form on your behalf. | Thank you, we have added this statement to the cover letter. |
| 3. We note that you have indicated that there are restrictions to data sharing for this study. PLOS only allows data to be available upon request if there are legal or ethical restrictions on sharing data publicly. For more information on unacceptable data access restrictions, please see [http://journals.plos.org/plosone/s/data-availability#loc-unacceptable-data-access-restrictions.](https://nam12.safelinks.protection.outlook.com/?url=https%3A%2F%2Furldefense.com%2Fv3%2F__http%3A%2F%2Fjournals.plos.org%2Fplosone%2Fs%2Fdata-availability*loc-unacceptable-data-access-restrictions.__%3BIw!!JmPEgBY0HMszNaDT!sArbiVI8lcMDYKo3S1KpXBoOGPUsT9WrXZWzubyjRnOz-kbbJeTTkM0B5uiprKanagtvyIm-V_V53ZyX_TQY%24&data=05%7C02%7Cclaire.richards%40wsu.edu%7C22187065e67f4fb841b208dc7b415568%7Cb52be471f7f147b4a8790c799bb53db5%7C0%7C0%7C638520768072037871%7CUnknown%7CTWFpbGZsb3d8eyJWIjoiMC4wLjAwMDAiLCJQIjoiV2luMzIiLCJBTiI6Ik1haWwiLCJXVCI6Mn0%3D%7C80000%7C%7C%7C&sdata=VBAURFE0kmGPGwJ5F%2BRaQbVXU3JztbvmctbdiyxyuKY%3D&reserved=0)  Before we proceed with your manuscript, please address the following prompts:  a) If there are ethical or legal restrictions on sharing a de-identified data set, please explain them in detail (e.g., data contain potentially identifying or sensitive patient information, data are owned by a third-party organization, etc.) and who has imposed them (e.g., a Research Ethics Committee or Institutional Review Board, etc.). Please also provide contact information for a data access committee, ethics committee, or other institutional body to which data requests may be sent.  b) If there are no restrictions, please upload the minimal anonymized data set necessary to replicate your study findings to a stable, public repository and provide us with the relevant URLs, DOIs, or accession numbers. For a list of recommended repositories, please see [https://journals.plos.org/plosone/s/recommended-repositories](https://nam12.safelinks.protection.outlook.com/?url=https%3A%2F%2Furldefense.com%2Fv3%2F__https%3A%2F%2Fjournals.plos.org%2Fplosone%2Fs%2Frecommended-repositories__%3B!!JmPEgBY0HMszNaDT!sArbiVI8lcMDYKo3S1KpXBoOGPUsT9WrXZWzubyjRnOz-kbbJeTTkM0B5uiprKanagtvyIm-V_V53SGcA2ib%24&data=05%7C02%7Cclaire.richards%40wsu.edu%7C22187065e67f4fb841b208dc7b415568%7Cb52be471f7f147b4a8790c799bb53db5%7C0%7C0%7C638520768072043395%7CUnknown%7CTWFpbGZsb3d8eyJWIjoiMC4wLjAwMDAiLCJQIjoiV2luMzIiLCJBTiI6Ik1haWwiLCJXVCI6Mn0%3D%7C80000%7C%7C%7C&sdata=VjBN6E3jxhrx9zirHLlSbhNBJeJwJRCwTx06u%2BFt%2BJ8%3D&reserved=0). You also have the option of uploading the data as Supporting Information files, but we would recommend depositing data directly to a data repository if possible.  We will update your Data Availability statement on your behalf to reflect the information you provide. | We have been given permission to share the processed dataset that was used for the analysis. Thus, the minimal anonymized data set has been included via the Fig Share Repository. The data restrictions apply to the raw, unprocessed data, though most publications on this topic do not include data or only include pre-processed data. For this reason, we believe that we are complying with PLOS ONE data access policies that state “…authors do not need to submit the raw data collected during an investigation if the standard in the field is to share data that have been processed.” Since we are compliant with the data sharing policies, we have revised the data sharing statement to note that the raw, unprocessed data can be purchased, without emphasizing that we are not sharing them. |
| 4. We note that Figure 2 in your submission contain [map/satellite] images which may be copyrighted. All PLOS content is published under the Creative Commons Attribution License (CC BY 4.0), which means that the manuscript, images, and Supporting Information files will be freely available online, and any third party is permitted to access, download, copy, distribute, and use these materials in any way, even commercially, with proper attribution. For these reasons, we cannot publish previously copyrighted maps or satellite images created using proprietary data, such as Google software (Google Maps, Street View, and Earth). For more information, see our copyright guidelines: [http://journals.plos.org/plosone/s/licenses-and-copyright](https://nam12.safelinks.protection.outlook.com/?url=https%3A%2F%2Furldefense.com%2Fv3%2F__http%3A%2F%2Fjournals.plos.org%2Fplosone%2Fs%2Flicenses-and-copyright__%3B!!JmPEgBY0HMszNaDT!sArbiVI8lcMDYKo3S1KpXBoOGPUsT9WrXZWzubyjRnOz-kbbJeTTkM0B5uiprKanagtvyIm-V_V53S5MeQ-B%24&data=05%7C02%7Cclaire.richards%40wsu.edu%7C22187065e67f4fb841b208dc7b415568%7Cb52be471f7f147b4a8790c799bb53db5%7C0%7C0%7C638520768072048928%7CUnknown%7CTWFpbGZsb3d8eyJWIjoiMC4wLjAwMDAiLCJQIjoiV2luMzIiLCJBTiI6Ik1haWwiLCJXVCI6Mn0%3D%7C80000%7C%7C%7C&sdata=TRBtGs39nxYn3JeXtHPYAlW7YCHIZ5WFJ2mqyYz6eDk%3D&reserved=0).  We require you to either (1) present written permission from the copyright holder to publish these figures specifically under the CC BY 4.0 license, or (2) remove the figures from your submission:   a. You may seek permission from the original copyright holder of Figure 2 to publish the content specifically under the CC BY 4.0 license.     We recommend that you contact the original copyright holder with the Content Permission Form ([http://journals.plos.org/plosone/s/file?id=7c09/content-permission-form.pdf](https://nam12.safelinks.protection.outlook.com/?url=https%3A%2F%2Furldefense.com%2Fv3%2F__http%3A%2F%2Fjournals.plos.org%2Fplosone%2Fs%2Ffile%3Fid%3D7c09*content-permission-form.pdf__%3BLw!!JmPEgBY0HMszNaDT!sArbiVI8lcMDYKo3S1KpXBoOGPUsT9WrXZWzubyjRnOz-kbbJeTTkM0B5uiprKanagtvyIm-V_V53fnV-QvN%24&data=05%7C02%7Cclaire.richards%40wsu.edu%7C22187065e67f4fb841b208dc7b415568%7Cb52be471f7f147b4a8790c799bb53db5%7C0%7C0%7C638520768072054563%7CUnknown%7CTWFpbGZsb3d8eyJWIjoiMC4wLjAwMDAiLCJQIjoiV2luMzIiLCJBTiI6Ik1haWwiLCJXVCI6Mn0%3D%7C80000%7C%7C%7C&sdata=U5oMo9a1wcXdaeEiX09f%2BMF7B5n7L7g8OLJSr5Wh1MY%3D&reserved=0)) and the following text: “I request permission for the open-access journal PLOS ONE to publish XXX under the Creative Commons Attribution License (CCAL) CC BY 4.0 ([http://creativecommons.org/licenses/by/4.0/](https://nam12.safelinks.protection.outlook.com/?url=https%3A%2F%2Furldefense.com%2Fv3%2F__http%3A%2F%2Fcreativecommons.org%2Flicenses%2Fby%2F4.0%2F__%3B!!JmPEgBY0HMszNaDT!sArbiVI8lcMDYKo3S1KpXBoOGPUsT9WrXZWzubyjRnOz-kbbJeTTkM0B5uiprKanagtvyIm-V_V53XXSIrZx%24&data=05%7C02%7Cclaire.richards%40wsu.edu%7C22187065e67f4fb841b208dc7b415568%7Cb52be471f7f147b4a8790c799bb53db5%7C0%7C0%7C638520768072060259%7CUnknown%7CTWFpbGZsb3d8eyJWIjoiMC4wLjAwMDAiLCJQIjoiV2luMzIiLCJBTiI6Ik1haWwiLCJXVCI6Mn0%3D%7C80000%7C%7C%7C&sdata=PZcGlQEyQ4Q93yTwLxInrS%2BFBtaFSmi%2BpNFxTf4Wi4E%3D&reserved=0)). Please be aware that this license allows unrestricted use and distribution, even commercially, by third parties. Please reply and provide explicit written permission to publish XXX under a CC BY license and complete the attached form.”   Please upload the completed Content Permission Form or other proof of granted permissions as an ""Other"" file with your submission.   In the figure caption of the copyrighted figure, please include the following text: “Reprinted from [ref] under a CC BY license, with permission from [name of publisher], original copyright [original copyright year].”   b. If you are unable to obtain permission from the original copyright holder to publish these figures under the CC BY 4.0 license or if the copyright holder’s requirements are incompatible with the CC BY 4.0 license, please either i) remove the figure or ii) supply a replacement figure that complies with the CC BY 4.0 license. Please check copyright information on all replacement figures and update the figure caption with source information. If applicable, please specify in the figure caption text when a figure is similar but not identical to the original image and is therefore for illustrative purposes only. The following resources for replacing copyrighted map figures may be helpful:   USGS National Map Viewer (public domain): [http://viewer.nationalmap.gov/viewer/](https://nam12.safelinks.protection.outlook.com/?url=https%3A%2F%2Furldefense.com%2Fv3%2F__http%3A%2F%2Fviewer.nationalmap.gov%2Fviewer%2F__%3B!!JmPEgBY0HMszNaDT!sArbiVI8lcMDYKo3S1KpXBoOGPUsT9WrXZWzubyjRnOz-kbbJeTTkM0B5uiprKanagtvyIm-V_V53RVfiXTO%24&data=05%7C02%7Cclaire.richards%40wsu.edu%7C22187065e67f4fb841b208dc7b415568%7Cb52be471f7f147b4a8790c799bb53db5%7C0%7C0%7C638520768072065683%7CUnknown%7CTWFpbGZsb3d8eyJWIjoiMC4wLjAwMDAiLCJQIjoiV2luMzIiLCJBTiI6Ik1haWwiLCJXVCI6Mn0%3D%7C80000%7C%7C%7C&sdata=%2BUX3cRI04eB29cGsCWgoukyv5oqH4B%2FyRpyI1HiKtfI%3D&reserved=0) The Gateway to Astronaut Photography of Earth (public domain): [http://eol.jsc.nasa.gov/sseop/clickmap/](https://nam12.safelinks.protection.outlook.com/?url=https%3A%2F%2Furldefense.com%2Fv3%2F__http%3A%2F%2Feol.jsc.nasa.gov%2Fsseop%2Fclickmap%2F__%3B!!JmPEgBY0HMszNaDT!sArbiVI8lcMDYKo3S1KpXBoOGPUsT9WrXZWzubyjRnOz-kbbJeTTkM0B5uiprKanagtvyIm-V_V53WCLZIbD%24&data=05%7C02%7Cclaire.richards%40wsu.edu%7C22187065e67f4fb841b208dc7b415568%7Cb52be471f7f147b4a8790c799bb53db5%7C0%7C0%7C638520768072071639%7CUnknown%7CTWFpbGZsb3d8eyJWIjoiMC4wLjAwMDAiLCJQIjoiV2luMzIiLCJBTiI6Ik1haWwiLCJXVCI6Mn0%3D%7C80000%7C%7C%7C&sdata=K1ZdysplQWjPpmh15Fm93%2BCQs69BIVeqXLc7MBD7v%2Fc%3D&reserved=0) Maps at the CIA (public domain): [https://www.cia.gov/library/publications/the-world-factbook/index.html](https://nam12.safelinks.protection.outlook.com/?url=https%3A%2F%2Furldefense.com%2Fv3%2F__https%3A%2F%2Fwww.cia.gov%2Flibrary%2Fpublications%2Fthe-world-factbook%2Findex.html__%3B!!JmPEgBY0HMszNaDT!sArbiVI8lcMDYKo3S1KpXBoOGPUsT9WrXZWzubyjRnOz-kbbJeTTkM0B5uiprKanagtvyIm-V_V53TzCNiob%24&data=05%7C02%7Cclaire.richards%40wsu.edu%7C22187065e67f4fb841b208dc7b415568%7Cb52be471f7f147b4a8790c799bb53db5%7C0%7C0%7C638520768072077523%7CUnknown%7CTWFpbGZsb3d8eyJWIjoiMC4wLjAwMDAiLCJQIjoiV2luMzIiLCJBTiI6Ik1haWwiLCJXVCI6Mn0%3D%7C80000%7C%7C%7C&sdata=iT46VILTkelxJ0ISSKIHR6%2FAhVwU1sO1ViHHdAz%2F3Ls%3D&reserved=0) and [https://www.cia.gov/library/publications/cia-maps-publications/index.html](https://nam12.safelinks.protection.outlook.com/?url=https%3A%2F%2Furldefense.com%2Fv3%2F__https%3A%2F%2Fwww.cia.gov%2Flibrary%2Fpublications%2Fcia-maps-publications%2Findex.html__%3B!!JmPEgBY0HMszNaDT!sArbiVI8lcMDYKo3S1KpXBoOGPUsT9WrXZWzubyjRnOz-kbbJeTTkM0B5uiprKanagtvyIm-V_V53fzI-Zxe%24&data=05%7C02%7Cclaire.richards%40wsu.edu%7C22187065e67f4fb841b208dc7b415568%7Cb52be471f7f147b4a8790c799bb53db5%7C0%7C0%7C638520768072083324%7CUnknown%7CTWFpbGZsb3d8eyJWIjoiMC4wLjAwMDAiLCJQIjoiV2luMzIiLCJBTiI6Ik1haWwiLCJXVCI6Mn0%3D%7C80000%7C%7C%7C&sdata=NP6UXf6OUbc1sbAB4YfA9kajo9QDvHYldVPcL5QEeeQ%3D&reserved=0) NASA Earth Observatory (public domain): [http://earthobservatory.nasa.gov/](https://nam12.safelinks.protection.outlook.com/?url=https%3A%2F%2Furldefense.com%2Fv3%2F__http%3A%2F%2Fearthobservatory.nasa.gov%2F__%3B!!JmPEgBY0HMszNaDT!sArbiVI8lcMDYKo3S1KpXBoOGPUsT9WrXZWzubyjRnOz-kbbJeTTkM0B5uiprKanagtvyIm-V_V53VHmdcmf%24&data=05%7C02%7Cclaire.richards%40wsu.edu%7C22187065e67f4fb841b208dc7b415568%7Cb52be471f7f147b4a8790c799bb53db5%7C0%7C0%7C638520768072089173%7CUnknown%7CTWFpbGZsb3d8eyJWIjoiMC4wLjAwMDAiLCJQIjoiV2luMzIiLCJBTiI6Ik1haWwiLCJXVCI6Mn0%3D%7C80000%7C%7C%7C&sdata=vK3ig7zy%2Bh98h%2ByjuDwF4%2F3zSxke%2BvIYGSvepY9evyA%3D&reserved=0) Landsat: [http://landsat.visibleearth.nasa.gov/](https://nam12.safelinks.protection.outlook.com/?url=https%3A%2F%2Furldefense.com%2Fv3%2F__http%3A%2F%2Flandsat.visibleearth.nasa.gov%2F__%3B!!JmPEgBY0HMszNaDT!sArbiVI8lcMDYKo3S1KpXBoOGPUsT9WrXZWzubyjRnOz-kbbJeTTkM0B5uiprKanagtvyIm-V_V53e-28cm2%24&data=05%7C02%7Cclaire.richards%40wsu.edu%7C22187065e67f4fb841b208dc7b415568%7Cb52be471f7f147b4a8790c799bb53db5%7C0%7C0%7C638520768072097116%7CUnknown%7CTWFpbGZsb3d8eyJWIjoiMC4wLjAwMDAiLCJQIjoiV2luMzIiLCJBTiI6Ik1haWwiLCJXVCI6Mn0%3D%7C80000%7C%7C%7C&sdata=3N98khhh79QNRPlMgRtbDTtRk%2Bnsp5iUicq%2BVDtdnPc%3D&reserved=0) USGS EROS (Earth Resources Observatory and Science (EROS) Center) (public domain): [http://eros.usgs.gov/#](https://nam12.safelinks.protection.outlook.com/?url=https%3A%2F%2Furldefense.com%2Fv3%2F__http%3A%2F%2Feros.usgs.gov%2F*__%3BIw!!JmPEgBY0HMszNaDT!sArbiVI8lcMDYKo3S1KpXBoOGPUsT9WrXZWzubyjRnOz-kbbJeTTkM0B5uiprKanagtvyIm-V_V53Y5HYjfX%24&data=05%7C02%7Cclaire.richards%40wsu.edu%7C22187065e67f4fb841b208dc7b415568%7Cb52be471f7f147b4a8790c799bb53db5%7C0%7C0%7C638520768072103383%7CUnknown%7CTWFpbGZsb3d8eyJWIjoiMC4wLjAwMDAiLCJQIjoiV2luMzIiLCJBTiI6Ik1haWwiLCJXVCI6Mn0%3D%7C80000%7C%7C%7C&sdata=NRDcGNciswZLWNUHt6DDD%2BEPJXJ8EMIflGAjdL1Ibyg%3D&reserved=0) Natural Earth (public domain): [http://www.naturalearthdata.com/](https://nam12.safelinks.protection.outlook.com/?url=https%3A%2F%2Furldefense.com%2Fv3%2F__http%3A%2F%2Fwww.naturalearthdata.com%2F__%3B!!JmPEgBY0HMszNaDT!sArbiVI8lcMDYKo3S1KpXBoOGPUsT9WrXZWzubyjRnOz-kbbJeTTkM0B5uiprKanagtvyIm-V_V53QkpOM7K%24&data=05%7C02%7Cclaire.richards%40wsu.edu%7C22187065e67f4fb841b208dc7b415568%7Cb52be471f7f147b4a8790c799bb53db5%7C0%7C0%7C638520768072109204%7CUnknown%7CTWFpbGZsb3d8eyJWIjoiMC4wLjAwMDAiLCJQIjoiV2luMzIiLCJBTiI6Ik1haWwiLCJXVCI6Mn0%3D%7C80000%7C%7C%7C&sdata=QLMUP4c1LSsgCqWUiuZRtCIKeWZkANO1SULF3ga2ceU%3D&reserved=0) | The county boundary shapefile is publicly available data. This was combined with the service territories (also publicly available) that were then revised based on utility maps.  We have revised the caption of the figure to include this additional information. |
| **Reviewer 1** | |
| **Comment** | **Action(s) performed** |
| This paper presents an analysis of the association between social vulnerability predictors, weather predictors, and power outage burden in Washington state. The major takeaways from this paper are that the relationship between social vulnerability factors and power outages are complex and nonlinear and that current research is limited by inconsistent and inaccurate outage data. The manuscript is well-written and technically sound. The authors perform rigorous statistical analyses and present their findings in a clear and detailed manner. The comparison between their approach of estimating customer counts and downscaling is helpful for future researchers in this area, as is their validation of PowerOutages.us datasets and DOE and EIA data. The discussion is particularly thorough and the authors did a great job of discussing their results in the context of relevant research. The paper could benefit from the addition of a conclusion section. | Thank you for the supportive comments! |
| Revisions include: - Please add a conclusion section to the manuscript. | We have added a conclusions section, thank you for the recommendation. |
| - Line 101 contains an incomplete sentence. "scale of POs. is more sensitive...". Please fix this. | Thank you for this suggestion. We fixed the beginning of the second sentence. |
| - Line 315, please define the acronym MNAR. Note that this is defined later on line 563. | This was fixed, and we removed the definition from its later use. |
| - Remove the period from the end of the section titles on lines 264 and 454 to remain consistent with the rest of the manuscript. | We removed the periods and made all section titles consistent--our apologies. |
| - Line 186: IEEE has already been defined in line 69 so there is no need to define it again. "IEE 1366-2005" should be changed to "IEEE 1366-2005". Please be more specific about the "other method" mentioned in line 187. | This is a good point. This is the language used in the EIA-861 instructions. We reached out to the EIA to clarify, and they explained that the utilities may follow similar practices as the IEEE methods but may not wish to certify to that standard. It is a bit ambiguous what it means or how it might affect the results utilities share. We clarified this within the manuscript in that section. |
| **Reviewer 2** | |
| **Comment** | **Action(s) performed** |
| This paper studies the relationships between power outages and social economic status of communities using data from Washington state. The paper consists of three aspects. First, the authors conduct data processing, with several more careful treatments that were not done by the prior work. Second, the paper proposes a model to relate power outages with social economic status of communities, i.e., Social Vulnerability Indices from CDC. Third, the paper uses the data to fit model parameters, and analyzes the results.  The strengths of the paper include careful and meticulous data processing. In particular, the authors separate major events from moderate disruptions using IEEE standards, and removing spurious data samples. Such efforts set the stage for data analysis. The model used appears to be different from those of the prior work. Overall, the study is relevant as Washington state suffers from frequent weather-induced power outages. Such a study has not been done in the prior work.  Weaknesses of the paper and suggestions include the following. | Thank you for the overview of the study. For clarification, this paper did not separate major events from other events, though we did remove spurious data, and describe the characteristics of major events according to several definitions used in prior studies of the health impacts of major power outages. We compared our SAIDI values estimated with the PowerOutage.us data with those reported by utilities that included the major events. However, this study models all outages, regardless of the size.  To address this, we added some additional explanations for why we used SAIDI and did not use thresholds for major events, including potentially spurious results and difficulty comparing across studies (in the introduction and discussion). |
| -First of all, the paper needs a major rewrite to be accessible by readers. For example, the paper can be structured to keep the key aspects of the study in the main text while moving the detailed implementation to the supplementary sections. For instance, the data section may keep what is needed for preprocessing and why that is important. How to do processing can be in the supplementary. | Thank you, we have moved much of the pre-processing into the supplementary material. |
| -The model equations, e.g., Equ (3) and (5) need to use standard and clear mathematical expressions. For example, what is the ``dependency” terms? Are covariate weighted linearly? Also, how to use the model also needs to be explained: are model parameters the same for all counties? Or the Bernoulli process pointwise, i.e., with different parameters for different counties? How are the model parameters estimated? Software used to implement the model can be moved to the supplementary. | Thank you for this suggestion. We have expanded these terms in the manuscript to answer these questions. The expectation of the response variable is the intercept plus the linear combination of smooth functions for each covariate.  The model parameters are the same for all county-utilities, except for the random effect for county-utility. |
| The model equations, e.g., Equ (3) and (5) need to use standard and clear mathematical expressions. For example, what is the ``dependency” terms? | We have expanded equations 3 and 5 to include all the covariates and the dependency terms. The dependency terms include the smoothers for the *JDay* and *DayInYear* and random effects for the county-utility. |
| Are covariate weighted linearly? | We do not know what you mean by weighted linearly, but wonder if you mean that the covariates are a linear combination, or if you are asking whether the covariates have a linear or non-linear effect? A GAM is a generalized linear model with a linear predictor involving a sum of smooth functions of covariates. In a GAM, covariates can have a non-linear effect (e.g., such as a plateau type relationship or trends that go up and down. Below are two references:  Wood SN. Generalized additive models: an introduction with R. 2nd ed. Chapman and Hall/CRC; 2017. doi:10.1201/9781315370279  Pedersen EJ, Miller DL, Simpson GL, Ross N. Hierarchical generalized additive models in ecology: an introduction with mgcv. PeerJ. 2019;7: e6876. doi:10.7717/peerj.6876 |
| Also, how to use the model also needs to be explained: are model parameters the same for all counties? | We are not doing random slopes, so slopes are constant over the county-utilities. However, the trends can change over the areas as we are applying hierarchical GAMs. For example, we allow for the temporal trends to differ by county-utility. |
| Or the Bernoulli process pointwise, i.e., with different parameters for different counties? | We are not familiar with a pointwise Bernoulli process, but we think this may be the same question as before… There is no interaction between covariates and county. |
| Software used to implement the model can be moved to the supplementary. | We prefer to reference the software and packages used for the analysis within the main manuscript rather than the supplementary materials as it is the standard practice within our fields. |
| How are the model parameters estimated? | The model parameters are estimated with numerical optimization of a likelihood criteria. See Wood 2017 for the mathematical details.  Wood SN. Generalized additive models: an introduction with R. 2nd ed. Chapman and Hall/CRC; 2017. doi:10.1201/9781315370279 |
| -Analysis of the results is now in ``discussion” section, which seems to be too long to be clear. To highlight the findings, the results and analysis can be a separate section. It needs to be made clear what key results are obtained, and how the results differ from the prior work (and why if known). For example, in what way, the outage durations are non-linear in SVI? Also, which part of the extensive data processing makes the difference to the results? Why are the impact of outages inconsistent in terms of SVIs? | We believe there may be confusion due to our section headings. We have made them the appropriate font size. Additionally, to make the results easier to navigate, we added third level headings (names of variables).  We have moved the summary of the results from the discussion section to the beginning of the results section, but still refer to some results within the discussion for comparison purposes.  We additionally added a note of the detail about what a GAMM does (lines 258-259 within the marked-up document) to help explain the non-linearity. The modeling approach indicates that the relationships are non-linear; otherwise, the edf would have been 1. This may be because of real non-linear relationships or because of other reasons, such as unaccounted for variables (e.g. physical factors) or the scale of analysis (aggregating the outage data on a county level). Whatever the reason for the non-linearity, a GAMM is required to avoid biased parameter estimates in a generalized linear mixed model (GLMM).  We agree that the terminology about the relationship between SVIs and outages being inconsistent is confusing and have removed this. |
| Some detailed comments:  -It will be more clear to specify the actual vertical axis in Figs 3, 4, 5, 6 (rather than ``partial effects”). | We included the term s(variable) to clarify this is the smoother function for the response variable and then included the term log-odds for the binomial. |
| -The paper uses DoE criterion for major events, i.e., outages that affected more than 50,000 (or >10,000) customers. Such a criterion is for transmission grid. For distribution grid, separating major events from moderate ones are considered in a prior work below.  Reference: Afsharinejad, A., et al., “Large-scale data analytics for resilient recovery services from power failures,” Joule Cell Press, 2021  Overall, this reviewer is positive about the paper but thought a revision is needed. | Thank you for this comment.  We agree that outage events can have different sizes and durations that are meaningful for public health, yet the DoE definition of major event can overlook them. This is why we did not set a criterion for major events, yet sought to provide a description of the major events using daily SAIDI values.  We appreciate the referenced paper, as it explores different ways to categorize outage events than have been referenced in health-related literature on outage events.  We intended to show it is possible to use a continuous outage exposure metric for studies of the health impacts of power outages. This work could be conducted to show the relationship between power outages and health impacts and define thresholds beyond which health impacts occur. Ideally, these metrics would involve CAIDI rather than SAIDI, but this would require more reliable outage data, with finer geographic resolution than currently available. |
